# Next generation of immune checkpoint inhibitors and beyond

**DOI:** 10.1186/s13045-021-01056-8

**Published:** 2021-03-19

**Authors:** Julian A. Marin-Acevedo, ErinMarie O. Kimbrough, Yanyan Lou

**Affiliations:** 1 Department of Hematology and Oncology, H. Lee Moffitt Cancer Center, Tampa, FL USA; 2 Division of Hematology and Oncology, Mayo Clinic, 4500 San Pablo Road, Jacksonville, FL 32224 USA

**Keywords:** Cancer, Immunotherapy, Tumor microenvironment, Immune evasion, Cytotoxic T lymphocytes, Immunotherapy, Immune checkpoint therapy, Inhibitory pathways

## Abstract

The immune system is the core defense against cancer development and progression. Failure of the immune system to recognize and eliminate malignant cells plays an important role in the pathogenesis of cancer. Tumor cells evade immune recognition, in part, due to the immunosuppressive features of the tumor microenvironment. Immunotherapy augments the host immune system to generate an antitumor effect. Immune checkpoints are pathways with inhibitory or stimulatory features that maintain self-tolerance and assist with immune response. The most well-described checkpoints are inhibitory in nature and include the cytotoxic T lymphocyte-associated molecule-4 (CTLA-4), programmed cell death receptor-1 (PD-1), and programmed cell death ligand-1 (PD-L1). Molecules that block these pathways to enhance the host immunologic activity against tumors have been developed and become standard of care in the treatment of many malignancies. Only a small percentage of patients have meaningful responses to these treatments, however. New pathways and molecules are being explored in an attempt to improve responses and application of immune checkpoint inhibition therapy. In this review, we aim to elucidate these novel immune inhibitory pathways, potential therapeutic molecules that are under development, and outline particular advantages and challenges with the use of each one of them.

## Background

Until recently, chemotherapy, radiation, and surgery were considered the cornerstones of cancer treatment. In 2011, with the approval of ipilimumab [1], immune checkpoint inhibitors were added to the therapeutic arsenal and revolutionized cancer management. These drugs not only introduced a new mechanism to treat cancer but also, in select cases, allowed for durable responses with a less toxic profile.

In contrast to old cytotoxic therapies, immune checkpoint inhibitors augment the host immune system to fight cancer. Under homeostatic conditions, there is a balance between pro-inflammatory and anti-inflammatory signaling maintained by immune checkpoints. These immune checkpoints are a set of inhibitory and stimulatory pathways that directly affect the function of immune cells [2]. Malignant cells disrupt this balance by promoting an immunosuppressive state that favors immune evasion and tumor growth [2, 3]. Cancer cells recruit regulatory T cells (Tregs), downregulate tumor antigen expression, induce T cell tolerance and/or apoptosis, and produce immune suppressive cytokines that stimulate inhibitory immune checkpoints [3]. This leads to a unique and highly immunosuppressive tumor microenvironment (TME) [4]. In an attempt to overcome these immunosuppressive conditions, immune checkpoint inhibitors act by blocking the effects of selected inhibitory pathways [2, 3, 5].

The best described inhibitory immune checkpoints are cytotoxic T lymphocyte-associated molecule-4 (CTLA-4), programmed cell death receptor-1 (PD-1), and programmed cell death receptor-1 ligand (PD-L1). T cell receptors (TCR) activate T cells. CTLA-4 is a molecule that is upregulated on the surface of active T cells to prevent excessive stimulation by the TCR. CTLA-4 competes with CD28, a TCR co-stimulatory receptor, to bind ligands like B7-1 and B7-2. This prevents CD28-mediated T cell activation [6]. PD-1 is also upregulated on activated T cells. PD-1 binds to its ligand, PD-L1, and transmits a negative costimulatory signal that limits T cell activation [6]. The oncogenic and immunosuppressive phenotype of the TME is characterized by overexpression of PD-L1 by cancer cells and overexpression of PD-1 and CTLA-4 by T cells [7]. Blockade of these molecules leads to immune-mediated anti-tumor response.

Ipilimumab, an anti-CTLA-4 monoclonal antibody, was the first FDA-approved immune checkpoint inhibitor and was used in patients with advanced melanoma [1]. Anti-PD-1 agents (nivolumab, pembrolizumab, cemiplimab) and anti-PD-L1 agents (atezolizumab, avelumab, durvalumab) were developed later [6]. These agents have been approved for use in multiple solid and hematologic malignancies [6]. They have improved treatment outcomes, and durable response has been seen even after discontinuation of therapy [8]. Their efficacy, however, is limited to a small number of patients [9].

In an attempt to improve response to therapy, combination strategies have been utilized. Anti-CTLA-4 agents have been used in conjunction with anti-PD-1/PD-L1 therapies. Although improved responses have been seen, the incidence and severity of toxicities is a concern [6, 7]. In particular, overactivation of the immune system leads to autoimmune-like side effects that can affect any organ and may require discontinuation of therapy, hospital admission, or management with systemic immunosuppressive drugs [10, 11].

New inhibitory checkpoints and their target molecules are being investigated to expand the use and efficacy of existing immune checkpoint inhibition therapy [12, 13]. In this review, we focus on these new investigational molecules (phase I and II clinical trials) and immune checkpoint inhibitory pathways that have emerged within the last 3 years. Table 1 compares these new investigational therapies to existing anti-CTLA-4, anti-PD-1, and anti-PD-L1 drugs. This is an update from a prior review of novel investigational molecules in immune checkpoint therapy published in 2018 [13].

**Table 1:** Existing immune checkpoint inhibitors and new immune inhibitory molecules

	Target	Agent	Mechanism of action	Indications	Advantages	Limitations
FDA-approved immune checkpoint inhibitors	CTLA-4	Ipilimumab	Inhibits CTLA-4 and allows T cell activation	CRC (in combination with nivolumab), HCC (in combination with nivolumab), melanoma (alone or in combination with nivolumab), mesothelioma (in combination with nivolumab), NSCLC (in combination with nivolumab), RCC (in combination with nivolumab)	Often better tolerated than chemotherapy - Used in a variety of solid and hematologic malignancies - Durable responses Potential for “cure” even in metastatic disease FDA-approved - Biomarkers available to predict response to therapy	Only a small proportion of patients benefit - Limited in cancers with “cold” TMEs - Autoimmune-like toxicities: - Cytopenias - Diarrhea/colitis - Fatigue - Hepatitis - Hypophysitis - Hypothyroidism - Myocarditis - Nephritis - Pneumonitis - Rash/pruritus - Uveitis
	PD-1	Cemiplimab	Inhibits PD-1 and allows T cell activation	BCC, CSCC, NSCLC		
		Nivolumab	Inhibits PD-1 and allows T cell activation	CRC (alone or in combination with ipilimumab), esophageal SCC, HCC (alone or in combination with ipilimumab), HL, HNSCC, melanoma (alone or in combination with ipilimumab), mesothelioma (in combination with ipilimumab), NSCLC (alone or in combination with ipilimumab), RCC (alone or in combination with ipilimumab), urothelial carcinoma		
		Pembrolizumab	Inhibits PD-1 and allows T cell activation	BC, cervical cancer, CRC, CSCC, endometrial carcinoma, esophageal carcinoma, gastric carcinoma, HCC, HL, HNSCC, melanoma, mesothelioma, MCC, MSI-High/MMR-deficient/TMB-high cancers, NSCLC, large B cell lymphoma, RCC, SCLC, urothelial carcinoma		
	PD-L1	Atezolizumab	Inhibits PD-L1 and allows T cell activation	BC, HCC, melanoma, NSCLC, SCLC, urothelial carcinoma		
		Avelumab	Inhibits PD-L1 and allows T cell activation	MCC, RCC, urothelial carcinoma		
		Durvalumab	Inhibits PD-L1 and allows T cell activation	NSCLC, SCLC, urothelial carcinoma		
New immune checkpoint inhibitors and other inhibitory targets	LAG-3 (CD223)	LAG525 (IMP701), REGN3767 (R3767), BI 754,091, tebotelimab (MGD013), eftilagimod alpha (IMP321), FS118	Inhibit LAG-3 and allow T cell activation	NA	Often better tolerated than chemotherapy - Can be used to enhance response to other ICIs - Responses seen in therapy refractory disease - Some may work in “cold” TMEs - Novel biomarkers available to further personalize treatment	Clinical outcomes not available for some agents - May not be potent enough to be used as monotherapy - Best combination strategies and indications are unclear - Use with other ICIs may increase toxicities - Toxicities may be similar to those found with the use of existing ICIs: - Cytopenias - Fatigue - Rash/pruritus - Diarrhea/colitis - Hepatitis - Pneumonitis - Unique toxicities and areas of concern: - Antigen sink (CD47) - Increased risk of infections - Hemolytic anemia (CD47) - Infertility (LIF) - Myositis - Neurotoxicity (SEMA4D) - On-target, off-tumor toxicities - Poor wound healing
	TIM-3	MBG453, Sym023, TSR-022	Inhibit TIM-3 and allow T cell activation			
	B7-H3, B7-H4	MGC018, FPA150	Inhibit B7-H3 or B7-H4 and allow T cell activation			
	A2aR	EOS100850, AB928	Inhibits A2aR and allow T cell and APC activation			
	CD73	CPI-006	Inhibit CD73 and allow T cell and APC activation			
	NKG2A	Monalizumab	Inhibits NKG2A and allows T cell activation			
	PVRIG/PVRL2	COM701	Inhibits PVRIG and allows T cell activation			
	CEACAM1	CM24	Inhibits CEACAM1 and allows T and NK cells activation			
	CEACAM 5/6	NEO-201	Inhibits CEACAM5 and 6 which allows T cell activation while interfering with tumor cell growth			
	FAK	Defactinib	Inhibits FAK and interferes with tumor growth			
	CCL2/CCR2	PF-04136309	Inhibits CCR-2 and allows T cell recruitment and activation			
	LIF	MSC-1	Inhibits LIF and allows T cell and APC activation while interfering with cancer growth			
	CD47/SIRPα	Hu5F9-G4 (5F9), ALX148, TTI-662, RRx-001	Inhibits CD47 or SIRPα and allows T cell and APC activation			
	CSF-1 (M-CSF)/CSF-1R	Lacnotuzumab (MCS110), LY3022855, SNDX-6352, emactuzumab (RG7155), pexidartinib (PLX3397)	Inhibits CSF-1 and allows APC activation			
	IL-1 and IL-1R3 (IL-1RAP)	CAN04, Canakinumab (ACZ885)	Inhibits IL-3 or IL-1RAP and allows T cell and APC activation			
	IL-8	BMS-986253	Inhibits IL-8 and decreases immunosuppressive TME while interfering with tumor growth			
	SEMA4D	Pepinemab (VX15/2503)	Inhibits SEMA4D and decreases immunosuppressive TME while interfering with tumor growth			
	Ang-2	Trebananib	Inhibits Ang-2 and allows APC activation while interfering with cancer growth			
	CLEVER-1	FP-1305	Inhibits CLEVER-1 and allows APC activation			
	Axl	Enapotamab vedotin (EnaV)	Inhibits Axl and allows APC activation while interfering with cancer growth			
	Phosphatidylserine	Bavituximab	Inhibits phosphatidylserine and allows T cell and APC activation while interfering with cancer growth			

BC, Breast cancer; BCC, basal cell carcinoma; CRC, colorectal cancer; CSCC, cutaneous squamous cell carcinoma; CTLA-4, cytotoxic T lymphocyte-associated molecule-4; HCC, hepatocellular carcinoma; HL, Hodgkin lymphoma; HNSCC, Head and neck squamous cell carcinoma; ICI, immune checkpoint inhibitor; MCC, Merkel cell carcinoma; MMR, mismatch repair; MSI, microsatellite instability; NSCLC, non-small cell lung cancer; NK, natural killer; PD-1, programmed cell death receptor-1; PD-L1, programmed cell death receptor-1 ligand; RCC, renal cell carcinoma; SCC, squamous cell carcinoma; SCLC, small cell lung cancer; TMB, tumor mutational burden; TME, tumor microenvironment

We conducted a PubMed search using the keywords and MeSH terms immunotherapy, immune checkpoint therapy, and immune checkpoint inhibitors. In addition, we used the American Society of Clinical Oncology (ASCO), American Association for Cancer Research (AACR) meeting abstracts and posters, and information from ClinicalTrials.gov. We included information from February 1, 2018, through June 1, 2020. We focused on phase I and phase II clinical trials using novel agents that block inhibitory immune checkpoints (e.g., LAG-3, TIM3) or pathways that act on other inhibitory immune mechanisms (e.g., CCL2/CCR2, IL-1, Ang2). Our data summarizes both preliminary results of ongoing trials, as well as completed clinical trials. We excluded phase III or later stage clinical trials, trials that explored well-described targets such as CTLA-4, PD-1, and/or PD-L1, immune stimulatory agents, vaccines, viruses, immune cellular therapy, and clinical trials involving the pediatric population. A total of 36 phase I, 9 phase I/II, and 7 phase II clinical trials were included in this review. A summary of the results can be found in Table 2.

**Table 2:** Summary of ongoing phase I, I/II, and II clinical trials utilizing immune checkpoint inhibition therapy

Category	Target	Drug	Trial	Phase	Type of tumor	Clinical efficacy	Safety	Comments
Inhibitory immune checkpoint targets	LAG-3 (CD223)	LAG525 (IMP701)	NCT02460224	I/II	Advanced malignancies	11/121 patients in the combination group achieved PR 1 patient had a CR	DLTs occurred in 4/121 patients included grade 3 and 4 pneumonitis, acute kidney injury, and autoimmune hepatitis	With or without spartalizumab
			NCT03365791	II	Solid or hematologic malignances	DCR for neuroendocrine tumors (86%), diffuse large B cell lymphoma (43%), and small cell lung cancer (27%)	11/72 patients had grade 3 or 4 AEs including dyspnea, fatigue, and poor appetite	In combination with spartalizumab
		REGN3767 (R3767)	NCT03005782	I	Solid or hematologic malignances	Monotherapy group: ORR 0% and DCR 48% with 12 SD Combination group: ORR 5% and DCR was 31% with 2 PR and 11 SD 2/12 PR and 6 SD in the group crossed over from monotherapy to the combination	1/67 DLT in the combination group (G4 CK elevation + G3 myasthenic syndrome + G1 elevation of troponin	Alone or in combination with cemiplimab
		BI 754,091	NCT03156114, NCT03433898, NCT03780725	I	Advanced or metastatic solid tumors	Not reported	21/321 DLTs, particularly infusion-related reactions (*n* = 6). Serious AEs: 77/321 (27%): pleural effusion (*n* = 6), deep venous thrombosis (*n* = 4), cardiac tamponade (*n* = 1), and acute kidney injury (*n* = 1)	Used in combination with anti-PD-1 therapy
			NCT03697304	II				
		Tebotelimab (MGD013)	NCT03219268	I	Advanced or metastatic solid or hematologic malignancies	Dose escalation (*n* = 29): ORR 10% and DCR 55% with 3 confirmed PR, 1 unconfirmed PR, and 13 SD Expansion cohort (*n* = 41): ORR 7%, DCR 59% with 3 PR, and 21 SD	2/207 DLTs: immune-mediated hepatitis and increased lipase	Alone or in combination with margetuximab (for patients who had expression of HER2 on their tumors)
		Eftilagimod alpha (IMP321)	NCT02676869	I	Advanced melanoma	1/18 CR	No DLTs reported	Used with pembrolizumab
			NCT03252938	I	Advanced solid tumors	ORR 17% and DCR 33% with 1/6 PR, 1/6 SD, and 4/6 PD	No DLTs reported	Used with avelumab
			NCT03625323	II	Advanced or metastatic NSCLC and HNSCC	ORR 47% and DCR 82% with 8/17 PR and 6/17 SD	Most common toxicities included cough (31%), fatigue (19%), and diarrhea (15%)	Used with pembrolizumab
		FS118	NCT03440437	I	Advanced solid tumors			Ongoing
	TIM-3	MBG453	NCT02608268	I/II	Advanced solid tumors	ORR in the monotherapy group was 0% and DCR was 29% with 25/87 SD ORR in the combination group was 5% and DCR was 44% with 4/86 PR and 34/86 SD	One DLT in combination cohort (grade 4 MG) 11% developed grade 3 or 4 AEs in the combination cohort	Combined with spartalizumab
		Sym023	NCT03489343	I	Advanced solid tumors and lymphomas	–	–	No results available
		TSR-022	NCT02817633	I	Advanced solid tumors	–	–	Ongoing
	B7-H3 and B7-H4	MGC018	NCT03729596	I/II	Advanced solid tumors	ORR 0% and DCR 15% with 3/20 SD	1 DLT: grade 4 neutropenia 3 serious AEs: pneumonitis, gastroenteritis, stasis dermatitis	Used as monotherapy
		FPA150	NCT03514121	I	B7-H4 positive solid malignancies	ORR 3% and DCR 38% with 1/29 PR and 10/29 SD	No DLTs or grade 4/5 toxicities were reported	Used as monotherapy
	A2aR	EOS100850	NCT02740985	I	Advanced solid tumors	ORR 0% and a DCR of 29% with 6/21 SD	No DLTs and no grade 3 or 4 AEs	Used as monotherapy. First in human
		AB928	NCT03719326 NCT03720678 NCT03629756	I	Advanced solid tumors	ORR 4% and DCR of 27% with 1/26 PR and 6/26 SD	1 DLT: grade 1 rash 6 patients with grade 3 or 4 AEs: fatigue, nausea, and cytopenias	Used in combination with standard chemotherapy or anti-PD-1 therapy
	CD73	CPI-006	NCT03454451	I	Advanced solid tumors	1 patient (monotherapy) with metastatic CRPC: substantial reduction in the size of a target lesion after only 5 cycles, sustained at the time of cutoff	No DLTs reported	Used as monotherapy or in combination with an anti-A2aR agent (CPI-444)
	NKG2A	Monalizumab	NCT03088059	II	Platinum-resistant, recurrent or metastatic, HNSCC	PFS: 7.4 weeks Median OS: 27.7 weeks ORR 0%, DCR 22% with 6/27 SD	No DLTs reported	Used as monotherapy
			NCT02643550	II	Platinum-resistant, recurrent or metastatic, HNSCC	ORR 20% DCR 58% with 8/40 PR 15/40 SD	No DLTs reported	Used in combination with cetuximab
	PVRIG/ PVRL2	COM701	NCT03667716	I	Advanced Solid tumors	DCR 57% (16/28 patients) No CRs 1/16 PR in the monotherapy group 1/12 unconfirmed PR in the combination group	No DLTs reported	Used as monotherapy and in combination with nivolumab
Inhibitory targets beyond immune checkpoints	CEACAM1	CM24	NCT02346955	I	Advanced or recurrent solid tumors	ORR of 0% and a DCR of 30% with 8/27 SD Median OS 4 months (lower dose), and 6 months (higher dose)	No DLTs 4 individuals with grade 3–4 GGT elevation	Used as monotherapy
	CEACAM 5/6	NEO-201	NCT03476681	I	Advanced solid tumors	ORR 0% and DCR 33% with radiological SD in 3/9 patients	No DLTs reported	Used as monotherapy
	FAK	Defactinib	NCT02546531	I	Advanced pancreatic adenocarcinoma	Escalation cohort (*n* = 8): ORR 13% and DCR 50% with 1 PR, 3 SD, and 4 PD Expansion cohort (= 20): ORR 5% and DCR 60% with 1 PR, 11 SD, 7 PD and 1 non-evaluable response	No DLTs were seen Most common grade 1 and 2 AEs included fatigue, anorexia, nausea, and vomiting	Used in combination with pembrolizumab and gemcitabine
	CCL2/ CCR2	PF-04136309	NCT02732938	I	Metastatic pancreatic adenocarcinoma	ORR 23.8%, DCR 38% with 0/21 CR, 5/21 confirmed PR, 1/21 unconfirmed PR, and 3/21 SD	DLTs: dysesthesia, hypokalemia, and hypoxia 24% pulmonary toxicities including 3 patients with grade 3 pneumonitis, 1 grade 4 hypoxia, and 1 grade 5 pneumonia	Used in combination with nab-paclitaxel and gemcitabine
	LIF	MSC-1	NCT03490669	I	Advanced solid tumors	DCR 22% with 9/41 SD lasting > 16 weeks	No DLTs reported	Used as monotherapy
	CD47/ SIRP	Hu5F9-G4 (5F9)	NCT02216409	I	Advanced Solid tumors	ORR ~ 5% DCR 19% with 2/43 PR (ovarian and fallopian tube cancers) and 6/43 SD (CRC)	AEs occurred with higher doses. These included constitutional symptoms (50%), headache (34%), anemia (39%), and lymphopenia (28%)	Used as monotherapy
			NCT02953509	I/II	Relapsed and refractory NHL	CRR 21% ORR 49% with 16/75 CR and 21/75 PR	DLTs 4% (no specifics provided)	Combined with rituximab
		ALX148	NCT03013218	I	Advanced solid tumors or refractory NHL	ICI-naïve HNSCC: ORR 40% (4/10), median PFS 4.6 months, and median OS not reached after 14 months of follow-up Non-ICI-naïve HNSCC: ORR 0%, median PFS 2 months, median OS 7.4 months ALX148 + trastuzumab in gastric/gastroesophageal cancers (*n* = 20): ORR 20%, median PFS 2.2 months, and median OS 8.1 months Monotherapy (*n* = 25): DCR 16% with 4/25 SD	2 DLTs: neutropenia with infection and thrombocytopenia with a significant bleed 1 grade 5 (fatal) toxicity under investigation Most AEs (66%) were low grade	Used in monotherapy agent or with pembrolizumab, trastuzumab, rituximab, ramucirumab, 5FU, paclitaxel, or cisplatin
		TTI-662	NCT03530683	I	Relapsed or refractory lymphomas	1 patient (DLBCL) with 5 prior lines of therapy achieved a PR by week 8 and a CR by week 36	No DLTs	Used as monotherapy
		RRx-001	NCT02518958	I	Advanced solid malignancies or lymphomas	ORR 25%, DCR 67% with 3/12 PR, 5/12 SD, and 3/12 PD	No DLTs reported 1 patient discontinued therapy due to pneumonitis	Used in combination with nivolumab
	CSF-1 (M-CSF)/ CSF-1R	Lacnotuzumab (MCS110)	NCT02807844	I/II	Advanced malignancies	DCR 27% 3/48 had pancreatic cancer: 1 PR, and 2 SD lasting > 300 days	No DLTs reported	Used in conjunction with spartalizumab
		LY3022855	NCT02265536	I	Metastatic BC and metastatic CRPC	BC (*n* = 22): DCR 23% with 5/22 SD. 2 of these had a response that lasted > 9 months CRPC (*n* = 7): ORR 0% and DCR 43% with 3/7 SD lasting up to 4 months	No DLTs reported	Used as monotherapy
		SNDX-6352	NCT03238027	I	Advanced solid tumors	DCR 13% with 4/32 SD that lasted > 4 months	2 DLTs, one grade 3 fatigue and one grade 3 pneumonitis	Used as monotherapy and in combination with durvalumab
		Emactuzumab (RG7155)	NCT01494688	I	Advanced solid tumors	Monotherapy (*n* = 99): ORR 0% and DCR 13% with 13/99 SD Combination (*n* = 54): ORR 7% DCR 50% with 4/54 PR 23/54 SD	No DLTs in the monotherapy, 2 DLTs in the combination: one grade 4 hypokalemia and one grade 3 hemorrhagic enterocolitis One grade 5 AE: bowel perforation	Used as monotherapy or in combination with paclitaxel
		Pexidartinib (PLX3397)	NCT01525602	I	Advanced solid tumors	ORR 16%, DCR 50%, PD rate 45% with 1/38 CR, 5/38 PR, 13/38 SD, and 17/38 PD	2 DLTs: one grade 3 atrial fibrillation and one grade 3 hypophosphatemia	Used in combination with paclitaxel
			NCT02777710	I	Advanced or metastatic pancreatic adenocarcinoma or CRC	ORR 0% and DCR 21% with 4/19 SD	2 DLTs: both transaminase elevation, one with hyperbilirubinemia	Used with durvalumab
			NCT02734433	I	Asian patients with symptomatic, advanced solid malignancies	DCR 67% with 1/11 PR and 4/11 SD	5 patients experienced at least one grade 3 or 4 AE: elevated transaminases and anemia	Monotherapy
	IL-1 and IL-1R3 (IL-1RAP)	CAN04	NCT03267316	I	Advanced or metastatic NSCLC, CRC, BC, or pancreatic adenocarcinoma	DCR 45% with 9/22 SD including 2 whose response lasted > 4 months	No DLTs or grade 4–5 AEs reported	Used as monotherapy
		Canakinumab (ACZ885)	NCT03968419	II	Early-stage NSCLC	–	–	Ongoing
	IL-8	BMS-986253	NCT02536469	I	Advanced solid tumors	ORR 0% and DCR 73% with 11/15 SD and 4/15 PD	No DLTs reported	Used as monotherapy
			NCT03400332	I/II	Advanced solid tumors	–	–	Ongoing
	SEMA4D	Pepinemab (VX15/2503)	NCT03268057	I/II	Advanced-stage NSCLC	Immunotherapy-naïve (*n* = 21): ORR 24% and DCR 81% with 5/21 PR and 12/21 SD Immunotherapy-refractory (*n* = 29): ORR was 7% and DCR was 59% with 2 patients achieving PR and 15 SD	No DLTs reported	Used in combination with avelumab
	Ang-2	Trebananib	NCT03239145	I	Advanced solid tumors	DCR 33% and ORR 7% with 1/15 PR and 4/15 SD Median time to progression: 2.6 months OS: 11.4 months	No DLTs and no grade 3 or 4 AEs	Used in combination with pembrolizumab
	CLEVER-1	FP-1305	NCT03733990	I/II	Advanced solid tumors	ORR 3% and DCR 27% with 2/30 PR, 6/30 SD, and 22/30 PD	No DLTs reported	Used as monotherapy
	Axl	Enapotamab vedotin (EnaV)	NCT02988817	I	Advanced solid tumors	ORR 6%, DCR 55% with 3/47 PR and 26/47 SD	6 DLTs: constipation, vomiting, GGT elevation, febrile neutropenia, and diarrhea	First-in-human clinical trial. Used as monotherapy
	Phosphatidylserine	Bavituximab	NCT01264705	II	Advanced, unresectable HCC	ORR 5%, DCR 58% with 2/38 PR and 20/38 SD	No DLTs or grade 4–5 AEs reported	Used in combination with sorafenib

AE, adverse event; BC, breast cancer; CK, creatine kinase; CR, complete response; CRC, colorectal cancer; CRR, complete response rate; CRPC, castrate-resistant prostate cancer; DCR, disease control rate; DLT, drug-limiting toxicities; GGT, Gamma-glutamyl transaminase; HCC, hepatocellular carcinoma; HNSCC, head and neck squamous cell carcinoma; ICI, immune checkpoint inhibitor; MG, Myasthenia Gravis; NHL, non-Hodgkin’s lymphoma; NSCLC, non-small cell lung carcinoma; OR, objective response; ORR, objective response rate; OS, overall survival; PD, progressive disease; PFS, progression-free survival; PR, partial response; SD, stable disease; SIRP, signal regulatory protein

## Inhibitory pathways

**Fig. 1:**
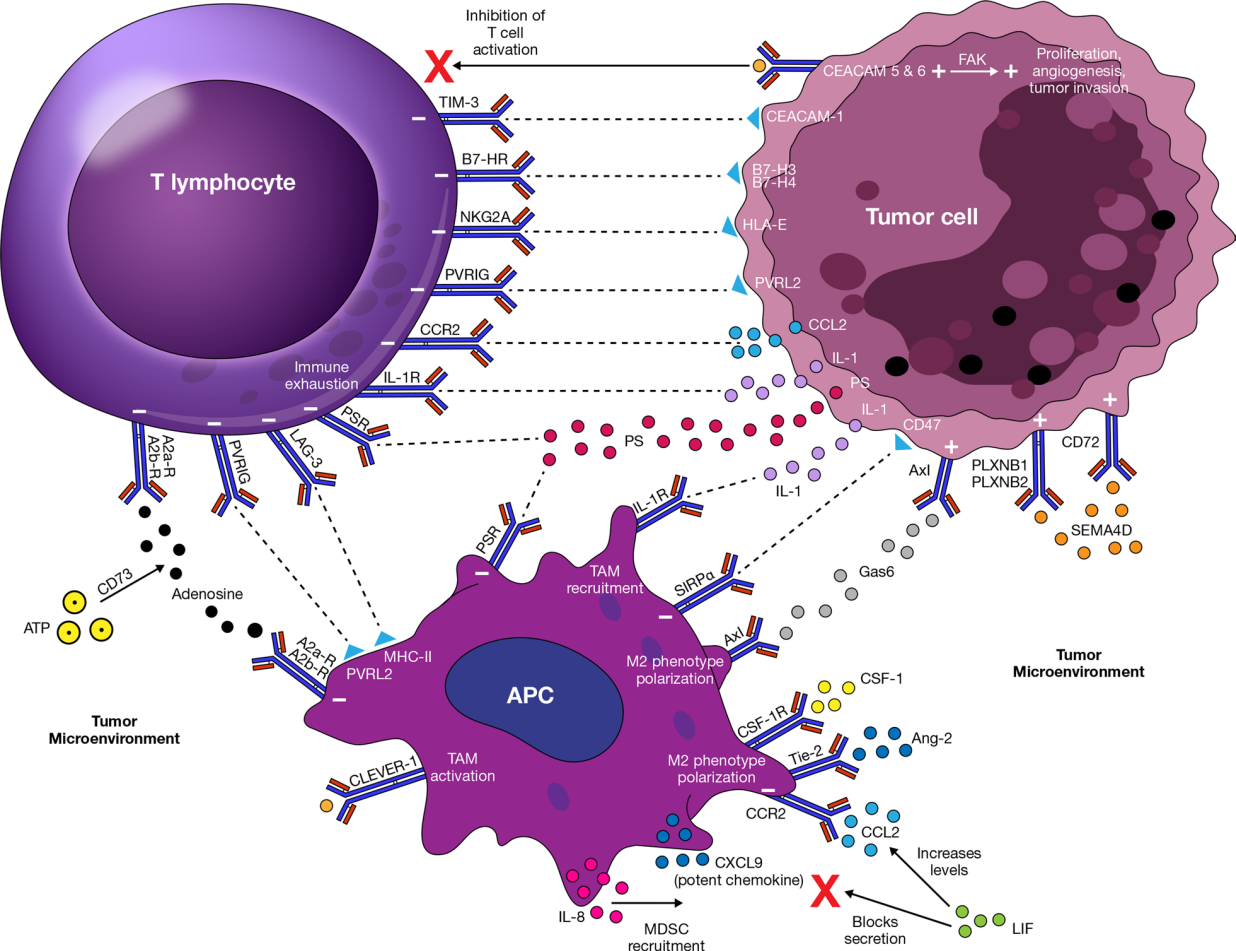
Inhibitory immune checkpoints and other inhibitory targets and their effects on immune-cell function and tumorigenesis

As mentioned previously, cell growth and immune evasion by malignant cells result from Treg recruitment, promotion of chronic inflammation and exhaustion of T cells, and expression of molecules like PD-L1 or CTLA-4, which induce a state of anergy among immune cells located in the TME [3, 7, 14, 15]. Other inhibitory molecules have been described. We classified these molecules as inhibitory immune checkpoints or inhibitory targets beyond immune checkpoints. This depends on whether the manipulation of the pathway has direct or indirect repercussions on T cell effects [13, 15, 16]. Figure 1 outlines the inhibitory pathways described below and their effects on immune-cell function and tumorigenesis.

## Inhibitory immune checkpoint targets

### LAG-3 (CD223)

Lymphocyte activation gene-3 (LAG-3, CD223) is a molecule that interacts with major histocompatibility complex (MHC) class II and is expressed by activated T cells, natural killers (NK) cells, B cells, and dendritic cells (DCs) [13, 17]. Although the mechanism of action of LAG-3 is incompletely understood, its interaction with MHC class II causes downregulation of T cell cytokine production, CD4 and CD8 T cell expansion, and favors Treg phenotype adoption to prevent tissue damage and autoimmunity [17] T cells located in the TME, known as tumor-infiltrating lymphocytes (TILs), overexpress LAG-3 which results in cell dysfunction, immune exhaustion, and favorable conditions for tumor growth [18]. Thus, LAG-3 blockade favors immune activation against malignant cells, while enhancing the effect of other immune checkpoint inhibitors (ICIs) (e.g., anti-PD-1 agents) and possibly other forms of immunotherapy [17, 19]. Combining LAG-3 inhibitors with other ICIs, however, could result in an increased incidence and severity of adverse events (AEs) [2]. Unfortunately, there are no biomarkers to predict who may benefit and who is likely to develop AEs from this therapy [19].

Six molecules are being investigated: five monoclonal antibodies (LAG525, REGN3767, BI 754111, tebotelimab, and FS118) and one LAG-3-Ig fusion protein (IMP321) as outlined below.

LAG525 (IMP701) is a monoclonal antibody (mAb) that targets LAG-3 and blocks the interaction with its ligand MHC class II. Preliminary data from a phase I/II clinical trial using LAG525 with or without spartalizumab in patients with advanced malignancies were published (NCT02460224) [20]. Out of 240 patients, 119 received LAG525 as monotherapy and 121 as combination therapy. Seventy-nine percent of patients receiving LAG525 monotherapy and 67% of patients on combination therapy discontinued therapy due to disease progression. Eleven of 121 patients in the combination group achieved a partial response (PR) and 1 patient had a complete response (CR). Data regarding response to monotherapy were not available [20]. Although the therapy was well tolerated, dose-limiting toxicities (DLTs) occurred in 4 patients in each arm and included grade 3 and 4 pneumonitis, acute kidney injury, and autoimmune hepatitis [20]. This trial has completed recruitment, and final data analysis is ongoing. Preliminary results of combination therapy revealed a 10% overall response rate (ORR [CR + PR]). However, it remains unclear whether this response was due to spartalizumab, LAG525, or both. This should be clarified once the finalized data are published. It would also be important to determine the efficacy of spartalizumab monotherapy in this setting.

Another phase II clinical trial investigated combination therapy with LAG525 and spartalizumab in patients with relapsed and/or refractory advanced solid or hematologic malignancies (NCT03365791) [21]. At the time of publication, 76 patients had been recruited, but only 72 were eligible for analysis. The primary endpoint was disease control rate (DCR [CR + PR + stable disease]). Preliminary results revealed a DCR that was particularly encouraging for neuroendocrine tumors (86%), diffuse large B cell lymphoma (43%), and small cell lung cancer (27%) [21]. The gastroesophageal cancer cohort was terminated because it did not reach the threshold for clinical benefit and was deemed futile [21]. No DLTs were mentioned. AEs affected 57% of all patients. Only 11/72 patients had grade 3 or 4 AEs including dyspnea, fatigue, and poor appetite [21]. This trial was completed, and final analysis is pending. Preliminary results suggest that LAG525 with spartalizumab may be effective for some but not all malignancies. Further research to identify patients who will benefit the most is warranted. In addition, it is important to determine whether the DCR seen was due to LAG525, spartalizumab, or combination therapy.

REGN3767 (R3767) is another mAb that targets LAG-3, blocking its interaction with MHC class II. A first-in-human phase I clinical trial using RGN3767 alone or in combination with cemiplimab in patients with advanced solid and hematologic malignancies who had progressed on prior lines of therapy is ongoing (NCT03005782). A total of 67 patients (25 in the monotherapy cohort and 42 in the combination group) with a median age 60–66 years were included [22]. In the monotherapy group, the ORR was 0% and  the DCR was 48%. There were no CR/PR and 12 patients achieved stable disease (SD). The ORR in the combination group was 5% and the DCR was 31%. Two patients achieved PR and 11 had SD [22]. There were 12 patients that crossed over from monotherapy to the combination arm. Two of these achieved PR and 6 SD. Overall, the drug was well tolerated with only 1 DLT in the combination group consisting of a grade 4 elevation of creatinine kinase associated with a grade 3 myasthenic syndrome and a grade 1 elevation of troponin. In addition, there was 1 case of grade 3 hypothyroidism in the combination group and 2 cases of grade 3 elevation of AST and ALT in the monotherapy group. The most common AEs were mild and included nausea in the monotherapy group and fatigue in the combination group [22]. Currently, the trial is still open and actively recruiting. Based on the available results, it appears that combination therapy with REGN3767 is more effective than monotherapy. Combination therapy, however, is more likely to result in severe toxicities. Future research should clarify which immunotherapy agent (cemiplimab or other) is best when combined with REGN3767.

BI 754091, another anti-LAG-3 mAb, is being tested in combination with anti-PD-1 therapy in three separate phase I clinical trials (NCT03156114, NCT03433898, NCT03780725) and one phase II clinical trial (NCT03697304). A review of the data from these trials was published and included here [23]. There were 321 patients with advanced or metastatic solid tumors included. The median age of patients was 63, and 62% (*n* = 200) were males. Although there is no mention of efficacy or clinical response, this medication showed an overall acceptable safety profile and was deemed similar to other ICIs. There were 21 cases of DLTs, particularly infusion-related reactions (*n* = 6). Serious AEs occurred in 77 patients (27%) including pleural effusion (*n* = 6), deep venous thrombosis (*n* = 4), cardiac tamponade (*n* = 1), and acute kidney injury (*n* = 1). Eighty-eight patients (30.9%) had grade 3 or 4 toxicities consisting of fatigue or immune-related AEs (irAEs). Although 86.7% experienced any AE, most were grade 1 and 2 and included fatigue (22.8%), fever (18.6%), or nausea [23]. The phase I trials are not actively recruiting patients. The phase II trial, however, is actively recruiting. While no efficacy data is available, results of these trials will help elucidate the role of anti-LAG3 therapy in combination with existing targets (anti-PD1 therapy). In addition, it will provide information regarding which combination strategy is most effective.

Tebotelimab (MGD013), a bi-specific mAb targeting both LAG-3 and PD-1, has been studied in a phase I clinical trial NCT03219268. This drug was used alone or in combination with margetuximab (for patients who had expression of HER2 on their tumors) in 207 patients with advanced or metastatic solid or hematologic malignancies [24]. Fifty of these patients were part of the dose-escalation cohort; 157 were included in the expansion cohort. Among the dose-escalation group, only 29 patients were response-evaluable. The ORR in this group was 10%, and DCR was 55% with 3 patients achieving a confirmed PR, 1 unconfirmed PR, and 13 SD [24]. In the expansion cohort, 41 patients were response-evaluable. The ORR in this group was 7% and DCR 59% with 3 cases of PR, and 21 with SD. Among 6 response-evaluable patients with HER2 expression who received margetuximab, 3 had PR [2 breast cancer (BC), 1 colorectal cancer (CRC)], and 2 SD [24]. There were 2 cases of DLTs including immune-mediated hepatitis and increased levels of lipase. AEs were reported in 146 patients (70.5%), but only 23.2% were grade ≥ 3 including rash, pancreatitis, and colitis. Most common grade 1–2 AEs were fatigue (19%) and nausea (11%) [24]. This trial is currently open for enrollment. Patients appear to respond to monotherapy with tebotelimab. This investigational drug will likely advance to subsequent phases of clinical trial. Five out of 6 HER2 positive patients had response. While the sample size was small, it raises the question of whether HER2 positivity might increase response to anti-LAG-3 therapy. It is also possible that the response seen was due to the anti-HER2 therapy.

FS118, a bi-specific antibody that targets LAG-3 and PD-L1, is being studied in a first-in-human phase I clinical trial in patients with advanced or metastatic solid malignancies who have failed prior anti-PD-1/PD-L1 therapy (NCT03440437). Recruitment was completed, but no results have been published to date. While no efficacy data are available, results of this trial will be important to help define the role anti-LAG-3 therapy when rechallenging patients who failed previous anti-PD-1/PD-L1 therapy.

Eftilagimod alpha (IMP321) is a soluble recombinant fusion protein that binds directly to MHC class II and blocks the interaction with LAG-3 on T cells. This molecule was tested in conjunction with pembrolizumab in 18 patients with advanced melanoma in a phase I clinical trial (NCT02676869) [25]. Fifty percent of patients showed a tumor reduction, but no specifics were provided. Of these, one patient achieved CR [25]. There were no DLTs reported nor were there any grade ≥ 4 toxicities. This trial is currently closed, and data analysis is ongoing. Those eligible for enrollment were actively receiving treatment with pembrolizumab and had not achieved a CR. The results will help assess the added benefit of anti-LAG-3 therapy in those patients with suboptimal response to anti-PD-1 therapy.

In another phase I clinical trial, subcutaneous eftilagimod alpha (IMP321) was combined with intravenous avelumab in 8 patients with advanced solid malignancies (NCT03252938) [26]. Preliminary results in 6 evaluable patients out of 8 patients demonstrated an ORR of 17% and DCR of 33% with 1 PR, 1 SD, and 4 progressive disease (PD). Overall, the therapy was well tolerated without DLTs. There was one grade 5 AE (acute kidney injury), no grade 4 AEs, and twelve grade 3 AEs, none of which were attributed to the study drug. Most AEs were grade 1 and 2 and included nausea, pain, and injection site reaction [26]. This trial is still active but is not currently recruiting. Final results will be important to assess the role of anti-PD-L1 therapy in combination with anti-LAG-3 therapy. In addition, it will help clarify which combination therapy is better tolerated and most effective. Of note, this trial will also assess for the safety of intratumoral and intraperitoneal use of eftilagimod alpha (IMP321) (NCT03252938).

Finally, a phase II clinical trial using eftilagimod alpha (IMP321) with pembrolizumab is also being performed in patients with advanced or metastatic non-small cell lung cancer (NSCLC) and head and neck squamous cell carcinoma (HNSCC) (NCT03625323). Preliminary results of 48 patients (73% males) with a median age of 66 years were published [27]. Among 17 patients with NSCLC who received eftilagimod alpha (IMP321) as first line, the ORR was 47% and DCR was 82% with 8 PR and 6 SD. Additionally, 6/15 patients (40%) with HNSCC who received eftilagimod alpha (IMP321) as second line and who had not received PD-1/PD-L1 therapy also achieved a PR [27]. The therapy was well tolerated, and only 3 patients discontinued treatment due to AEs. The most common toxicities included cough (31%), fatigue (19%), and diarrhea (15%) [27]. This trial is active and recruiting patients. While the sample size was limited, the clinical response to therapy appeared promising. The conclusion of this trial will help determine the added benefit of upfront anti-LAG-3 therapy to anti-PD-1 therapy. In addition, subset analysis may help define the role of anti-LAG-3 therapy in those patients previously treated with anti-PD-1/anti-PD-L1 agents.

### TIM-3

T cell immunoglobulin-3 (TIM-3) is an immune checkpoint that promotes immune tolerance. It is a receptor expressed by multiple cells including effector T cells, Tregs, B cells, macrophages, NK cells, DCs and even tumor cells [28, 29]. The main ligands include galectin-9, phosphatidyl serine, and carcinoembryonic antigen-related cell adhesion molecule (CEACAM)-1 [18]. TIM-3 stimulation by its ligands favors T cell exhaustion and promotes the expansion of myeloid-derived suppressor cells (MDSCs) in the TME, which facilitates tumor growth [29]. High TIM-3 levels have correlated with poor prognosis in various malignancies (e.g., prostate, renal cell, colon, cervical) [29, 30]. TIM-3 blockade results in decreased MDSCs and increased proliferation and cytokine production by T cells [29, 30]. Given its expression in a variety of T cells and its synergistic effects with other anti-PD-1 agents, TIM-3 blockade has become a particularly attractive target [28, 29]. The synergism may, however, increase the incidence and severity of irAEs. TIM-3 also plays a role in immune defense against organisms such as listeria and mycobacteria. Its blockade could result in an increased risk of these infections [29, 30].

An IgG4 mAb against TIM-3 (MBG453) was investigated alone and in conjunction with spartalizumab in a phase I/II clinical trial in patients with metastatic solid malignancies (NCT02608268). Out of 173 patients recruited, 87 received monotherapy and 86 received combination therapy [31]. The ORR in the monotherapy group was 0% and DCR was 29% with 25 patients achieving SD (four had received anti-PD-1/PD-L1 therapy). In the combination group, ORR was 5% and DCR was 44% with 4 patients achieving a PR (one which had been exposed to prior anti-PD-1/PD-L1 therapy), and 34 SD (ten had been exposed to prior anti-PD-1/PD-L1 therapy) [31]. There was one DLT reported in the combination cohort (grade 4 myasthenia gravis). Reported AEs were mostly grade 1 and 2 with no grade 3 or 4 in the monotherapy cohort and only 11% in the combination cohort. The most common AE was fatigue [31]. This trial is active but no longer recruiting. Both DCR and ORR were higher in the combination group. This suggests that anti-TIM-3 therapy may be a good adjunct therapy. Additional trials are needed to determine the benefit gained with addition of anti-TIM-3 agents to other existing immune therapies.

Sym023 and TSR-022 are two additional monoclonal antibodies targeting TIM-3 that are being investigated in two phase I clinical trials in patients with advanced solid tumors and lymphomas (NCT03489343, NCT02817633). No preliminary results are available. The Sym023 trial is now completed, and analysis is ongoing. The results of this trial will provide clarity on the efficacy of single agent anti-TIM-3 therapy. The TSR-022 trial is active and recruiting. This anti-TIM-3 therapy is being used with other investigational agents, nivolumab, or docetaxel. Results will provide information regarding the benefit of anti-TIM-3 agents combined with anti-PD-1 or chemotherapy agents. This will also help determine which combination strategy should be used.

### B7-H3 and B7-H4

B7 molecules are a family of transmembrane proteins that interact with CD28 receptor family and modulate either stimulatory or inhibitory immune signals [32, 33]. B7-H3 (CD276) is a member of the B7 family and is expressed in different solid organs including the spleen, liver, and heart. It is also expressed in immune cells such as Tregs, DCs, NKs, B cells, and T cells. Although B7-H3 was thought to be an immune stimulator, more recently it has been found to be an immunosuppressor. It dampens T cell activation, proliferation, and cytokine production and favors tumor progression [32, 33]. B7-H3 levels can be elevated in various hematologic and solid malignancies. Elevated levels correlate with poor prognosis in patients with NSCLC, renal cell carcinoma (RCC), and CRC [32, 33].

B7-H4 (B7S1, B7x, or Vtcn1), like B7-H3, is ubiquitously expressed by solid organs like the brain, kidney, liver, and spleen as well as immune cells particularly tumor-infiltrating antigen-presenting cells (APCs) [34]. Although its biological effect remains controversial, it appears to be mostly anti-inflammatory since it inhibits T cell activation and favors Tregs recruitment [34]. B7-H4 levels are elevated in different malignancies (e.g., lung, melanoma, RCC, CRC) and levels correlate with worse outcomes [34].

Anti-B7-H3 and anti-B7-H4 agents enhance T cell activation and promote cytotoxic activity and cytokine release without a significant increase in irAEs. The favorable side effect profile is thought to be due to a relatively low expression of B7-H3 and B7-H4 in normal tissues compared to the TME [32–34]. Better understanding of B7-H3/B7-H4 and its contribution to tumor growth, invasion, and immune evasion is required in order to develop better molecules and biomarkers to utilize these pathways [32].

MGC018, a duocarmycin-based antibody drug conjugate (ADC) targeting B7-H3, was investigated as monotherapy in a phase I/II clinical trial in 20 patients with advanced solid malignancies (NCT03729596) [35]. Results revealed an ORR of 0% and a DCR of 15% with 3 patients achieving SD. These three patients had a substantial reduction in their target lesions. One patient with small cell lung cancer (SCLC) had a 6% reduction, one patient with NSCLC had a 24% reduction, and one metastatic castrate-resistant prostate cancer (CRPC) patient had a 29% reduction in tumor size [35]. Additionally, a metastatic CRPC patient had a substantial improvement in his bone scan and PSA levels [35]. Unfortunately, the drug was toxic. Sixteen patients (80.9%) experienced at least one AE, 11 of them with at least a grade 3 AE. There were 3 serious AEs (pneumonitis, gastroenteritis, stasis dermatitis) and one DLT (grade 4 neutropenia). Other common AEs included leukopenia, skin toxicity, and infusion reactions [35]. The trial is ongoing and recruiting. Clinical responses were limited; however, three patients did derive benefit. It would be interesting to evaluate the characteristics of these tumors that could explain the response. For example, if pre-treatment B7-H3 levels were elevated in responders, perhaps these levels could be used as a biomarker for patient selection. Lastly, clinical application of this therapy may be limited due to the high incidence and severity of toxicities.

FPA150, a mAb targeting B7-H4, has been evaluated in a phase I clinical trial in patients with B7-H4 positive solid malignancies (NCT03514121). Recent reports from 29 patients (median age 63) revealed an ORR 3% and DCR of 38% with 1 PR and 10 SD. The PR patient had platinum-resistant ovarian cancer and had received treatment with seven lines of therapy and anti-PD-1 therapy [36]. No DLTs or grade 4/5 toxicities were reported. AEs were seen in 18/29 patients with only two grade 3 AEs (lymphopenia and hypertension). The rest were grade 1 and 2 and included fatigue, decreased appetite, and diarrhea [36]. This trial is currently active but not recruiting patients. The clinical responses are encouraging with acceptable toxicity profile. Additional research is needed to validate the use of B7-H4 as a biomarker for patient selection. It would also be interesting to assess whether other factors affect the response in spite of high expression of B7-H4.

### A2aR and CD73

Adenosine, as a component of adenosine triphosphate (ATP), mediates multiple physiologic and metabolic pathways. Extracellular levels are usually low in normal tissues. Adenosine levels increase dramatically in response to injury in an attempt to suppress excessive inflammation and allow for wound healing [37]. These effects are mediated by adenosine receptors including A2aR and A2bR. These receptors are expressed on multiple immune cells including T cells, APCs, neutrophils, and NK cells in which adenosine causes inhibitory effects [38]. Unlike normal tissue, TMEs express high levels of ATP as a consequence of tissue destruction, hypoxia, and inflammation. Catabolism of ATP is mediated by CD73, an enzyme that is normally expressed in tissues but overexpressed by MDSCs, tumor-associated macrophages (TAMs), Tregs, exhausted T cells, and tumor cells in the TME. ATP catabolism leads to high concentrations of extracellular adenosine which results in immune suppression, cell exhaustion, and tumor growth [37]. High levels of CD73 have been found in multiple malignancies and are associated with an overall poor prognosis [39].

Several novel agents targeting these pathways are under investigation in clinical trial. One potential advantage of this therapy is its ability to be used in combination with other anti-adenosine agents that target different steps (e.g., A2aR with anti-CD73) and/or combination with other types of immunotherapy. The main limitations with these agents include their short half-lives, limited efficacy when used as monotherapy, and uncertainty regarding best combination approaches [40].

EOS100850 is an oral ICI that directly binds and inhibits A2aR expressed by T-lymphocytes. It is being evaluated as monotherapy in a first-in-human phase I clinical trial in patients with refractory solid malignancies (NCT02740985). Preliminary results of 21 patients demonstrated an ORR of 0% and a DCR of 29% with 6 patients achieving SD [41]. Additionally, there were no DLTs and no grade 3 or 4 AEs. The most common toxicities included grade 1 and 2 nausea, vomiting, fatigue, and elevation of liver enzymes [41]. This trial is active but not enrolling patients at this time. Use of this agent as monotherapy resulted in limited activity, however, was well tolerated. Further evaluation of this therapy in combination with other agents should help determine whether improved response can be achieved. The oral administration of this drug is particularly attractive.

AB928 is an oral therapy with the ability to bind and inhibit both A2aR and A2bR on immune cells. Three phase I clinical trials are testing AB928 in combination with standard chemotherapy or anti-PD-1 therapy in patients with advanced or recurrent solid malignancies including triple-negative breast cancer (TNBC), ovarian cancer, gastroesophageal cancer, and CRC (NCT03719326, NCT03720678, NCT03629756). Recently, published results of 26 patients from all three trials show an ORR of 4% and a DCR of 27% with 1 patient achieving a PR (ovarian cancer) and 6 SD (all in the group receiving anti-PD-1 therapy) [42]. There was one case of DLT consisting of a grade 1 rash, and 6 additional patients developed grade 3 or 4 AEs including fatigue, nausea, and cytopenias [42]. The most common AEs were grade 1–2 including nausea, fatigue, vomiting, and elevated transaminases [42]. The NCT03719326 trial is actively recruiting, but the other two are no longer enrolling patients. Preliminary results of these trials are encouraging; however, ongoing investigation is needed to determine the role of anti-A2aR and anti-A2bR as adjunct therapy. It would be interesting to compare the results of combination therapy to chemotherapy or anti-PD-1 therapy alone.

CPI-006, a mAb directed against CD73, is being studied as monotherapy or combination therapy with an anti-A2aR agent (CPI-444) in a phase I clinical trial in patients with relapsed and incurable solid malignancies (NCT03454451). Preliminary results for 17 patients (11 monotherapy, 6 combination therapy), predominantly male (10 in the monotherapy cohort, 6 in the combination), and with a median age of 62–64 were recently published [43]. One patient in the monotherapy group with widely metastatic CRPC had a substantial reduction in the size of a target lesion after only 5 cycles, and this response was sustained at the time of cutoff for the data report. Although no other efficacy reports were available, there was a substantial increase in the effector T cell-to-Tregs ratio [43]. Therapy was well tolerated with no DLTs and a few grade 1 infusion reactions that were easily controlled with NSAIDs [43]. This trial is actively recruiting. The data available is limited to one patient; however, the patient appears to have had a robust response. It will be interesting to assess whether others have similar results. The use of T cell-to-Tregs ratio as a biomarker of response to other adenosine-associated pathways (anti-A2aR and anti-A2bR therapies, for example) could be considered.

### NKG2A

Natural killer group protein 2A (NKG2A) is a cell surface receptor and member of the NKG2 family. It is present on approximately 50% of circulating NK cells and on about 5% of circulating CD8 + T cells [44]. These levels substantially increase with chronic antigen exposure and under chronic inflammatory conditions [44]. Upon activation by its ligand HLA-E, a nonclassical MHC class I molecule, NKG2A dimerizes with CD94 and triggers a cascade of intracytoplasmic tyrosine-based inhibitory signals that suppress T and NK cell effector function [45]. Virally infected cells, for example, downregulate HLA-E favoring NK and T cell activation and antiviral responses [46]. In contrast, cancer evades the immune system by overexpressing HLA-E as well as recruiting TILs with high NKG2A/CD94 expression [45, 47]. High NKG2A expression correlates with worse survival in ovarian and colon cancer [45, 47].

Blockade of NKG2A enhances antitumor response by T and NK cells. However, currently available data suggest that monotherapy may be insufficient to achieve anti-tumor effects [45]. Thus, combination therapy is a more promising strategy to enhance other treatments like anti-PD-1/PD-L1 or anti-EGFR agents [44, 45]. The most effective combination strategy has not yet been elucidated.

Monalizumab, a humanized mAb targeted against NKG2A, was studied as monotherapy in a phase II clinical trial in patients with platinum-resistant, recurrent or metastatic, HNSCC (NCT03088059). Results of 27 patients (median age of 62), 16 (59%) of which had been exposed to anti-PD-1/PD-L1 agents, were recently published [48]. Specific cancers included oral cavity (26%), oropharynx (41%), hypopharynx (26%), and larynx (7%). Median progression-free survival (PFS) was 7.4 weeks, and median overall survival (OS) was 27.7 weeks. ORR was 0% and DCR was 22% with no objective responses and 6/27 patients with SD [48]. The study was terminated early because it did not meet its primary endpoint, objective response [48]. The safety profile was acceptable, and none of the grade 3 or higher toxicities were attributed to this drug [48]. The trial results presented confirm the limited clinical efficacy of anti-NKG2A therapy when used alone. While this arm of the trial was terminated early, the combination arm is currently open and enrolling patients.

In a separate phase II clinical trial, monalizumab is being used in combination with cetuximab in patients with platinum-resistant, recurrent or metastatic, HNSCC who have received 2 or fewer lines of therapy (NCT02643550). Recently published results of 40 patients revealed an ORR of 20% and a DCR of 58% with a total of 8 patients achieving PR and 15 SD [49]. After a median follow-up of 7.3 months, the median time to response was 1.6 months. Therapy was well tolerated according to previously published results from the same group [50]. Most AEs were grade 1–2 and were easily treated; however, no further details were provided [50]. This trial is currently enrolling patients. The results demonstrate the role of anti-NKG2 therapy as an adjunct treatment. It would be important to compare patients treated with combination therapy to those receiving cetuximab alone. Further research is needed to assess its use with other immune therapies.

### PVRIG/PVRL2

Poliovirus receptor-related immunoglobulin domain containing (PVRIG), also known as CD112R, is a recently described protein and member of the immunoglobulin superfamily receptors. It is expressed by CD4 + /CD8 + T and NK cells [51]. Its ligand, poliovirus receptor-related 2 (PVRL2, also known as CD112 and nectin-2), is expressed by DCs under normal conditions. PVRIG interferes with T cell activation, cytokine secretion, and expansion once bound with its ligand [51]. This pathway is often upregulated in cancer and TMEs. PVRIG is overexpressed particularly in CD4 + and CD8 + TILs in ovarian, breast, endometrial, lung, and kidney cancers [52]. PVRL2 can also be overexpressed in different malignancies including ovarian, prostate, and endometrial cancers [52]. Blockade of the PVRIG/PVRL2 pathway is attractive because its effects are independent of the PD-L1 pathway. This serves as an alternative therapeutic approach for individuals who lack PD-L1 expression or whose tumors are refractory to anti-PD-1/PD-L1 therapy [52]. Given its relatively recent discovery, it is unclear whether these agents will be potent enough to be used alone or whether they are more effective in conjunction with existing therapies.

COM701, a first-in-class mAb targeting PVRIG, is being studied in a phase I clinical trial in patients with advanced or metastatic solid malignancies refractory to standard therapies (NCT03667716). Recent results of 28 patients (16 treated with monotherapy and 12 in combination with nivolumab) demonstrated a DCR of 57% (16/28 patients) [53]. There were no CRs. There was 1 confirmed PR in the monotherapy group in a patient with primary peritoneal cancer who had received therapy for over 15 weeks. There was 1 additional patient in the combination group who achieved an unconfirmed PR and had been on therapy for over 34 weeks [53]. There were no DLTs reported, and the most common AEs were grade 1 and 2 fatigue, rash, edema, and nausea [53]. The trial is active and enrolling. There are limited data available; however, the results suggest potential benefit of this therapy without significant side effects. It may not only have a role as an adjunct therapy but could also become a viable alternative stand-alone treatment.

## Inhibitory Targets Beyond Immune Checkpoints

### CEACAM1, CEACAM5, CEACAM6, and FAK

CEACAM is a family of proteins that mediate different physiological effects ranging from tissue organization and angiogenesis to immune modulation [54]. CEACAM1 serves as a ligand of TIM-3 and inhibits the function of NK and T cells [18, 55]. This molecule is expressed by normal tissue, and it is often overexpressed in malignancies [55].

CEACAM5 serves as an adhesion molecule and is widely expressed by normal tissue. It has been found in various malignancies including breast, lung, gastrointestinal, and genitourinary cancers. It plays a role in inhibition of cell differentiation, inhibition of apoptosis, and interference with normal tissue architecture development [56]. It also interacts with CEACAM1 to inhibit NK-mediated killing, release of inflammatory cytokines, and interferes with the functioning of TILs [57]. CEACAM5 serves as a tumor marker, particularly in CRC [56].

CEACAM6 (CD66c) is expressed by healthy tissue and immune cells. It assists with tissue architectural organization and immune modulation including neutrophil adhesion and activation [58]. In malignant cells, it promotes proliferation, angiogenesis, tumor invasion, and immune suppression by interfering with myeloid and T cell activation [54, 56]. Importantly, CEACAM6 stimulation leads to the activation of various signaling pathways including FAK, an important driver in the switch to an invasive phenotype in cancer cells [56].

A theoretical advantage of targeting CEACAM proteins is the dual antitumor effect by directly interfering with tumor cell proliferation and invasion, while enhancing the immune system against cancer. A limitation of its use, however, includes CEACAM’s effects on neutrophil adhesion and activation [58]. Additionally, the lack of expression of CEACAM family proteins in mice has limited the ability to test these agents in the preclinical, animal setting [56].

CM24, a recombinant humanized mAb directed against CEACAM1, is being studied as monotherapy in a phase I clinical trial in patients with advanced or recurrent solid malignancies (NCT02346955). Results available for 27 patients (13 males, 14 females) with a median age of 60 demonstrated an ORR of 0% and a DCR of 30% with 8 patients achieving SD [59]. The median OS was 4 months in the low-dose group compared to 6.2 months in patients who received higher doses. This suggests that response may be dose-dependent. There were no reported DLTs. The most severe AE was grade 3–4 gamma-glutamyl transferase (GGT) elevation seen in 4 individuals. Most common AEs were grade 1 and 2, particularly elevation in transaminases (7 patients) [59]. The study was terminated for unclear reasons. Publication of the final results is still pending. While it appears the drug was well tolerated, there were no reports of PR or CR. Perhaps the use of this agent in combination with other therapies may enhance its efficacy.

NEO-201, a humanized mAb that targets CEACAM5 and CEACAM6, is being studied as monotherapy in a phase I clinical trial in patients with CEACAM5/6 positive, advanced solid malignancies (NCT03476681). Safety and pharmacokinetic data for this drug were recently published [60, 61]. Among 9 patients studied, the ORR was 0% and DCR was 33% with radiological SD seen in 3 patients. The remaining 6 patients experienced radiologic PD after 2 cycles. Those patients with SD were found to have low-serum CEACAM5 and low NK cell expression of CEACAM1. The opposite was true in those with PD [60]. The authors concluded that low NK CEACAM1 expression and low-serum CEACAM5 expression correlated with clinical response to this agent [60, 61]. NEO-201 was overall well tolerated with mild infusion reactions seen in all patients, and moderate fatigue seen in 3 of them [60, 61]. This study is actively enrolling. Preliminary results did demonstrate modest clinical efficacy; however, as outlined above perhaps combination therapy could improve response. NK CEACAM1 and serum CECEAM5 expression could represent new biomarkers to determine response to anti-CEACAM therapy. Additional trials are needed to validate these findings.

Defactinib, an oral tyrosine kinase inhibitor of FAK, is being used in conjunction with pembrolizumab and gemcitabine in patients with advanced pancreatic adenocarcinoma in a phase I clinical trial (NCT02546531). A total of 28 patients were evaluated and divided into dose-escalation phase (*n* = 8) and expansion cohort (*n* = 20). In the dose-escalation cohort, ORR was 13% and the DCR was 50% with 1 PR, 3 SD, and 4 PD. In the expansion cohort, the ORR was 5% and the DCR was 60% with 1 PR, 11 SD, 7 PD, and 1 had a non-evaluable response [62]. The median duration of treatment was 4.6 months. No DLTs were seen and the most common grade 1 and 2 AEs included fatigue, anorexia, nausea, and vomiting [62]. This trial is active but not recruiting. While the preliminary results did demonstrate efficacy, it is hard to determine the role anti-FAK therapy played given the concurrent use of pembrolizumab and gemcitabine. Further research is needed to determine the benefit of anti-FAK therapy with or without immune and chemotherapeutic agents. In addition, it would be interesting to assess the effects of anti-FAK therapy in combination with anti-CEACAM therapy.

### CCL2/CCR2

Chemokines promote migration, recruitment, differentiation, and activation of immune cells, including T effector cells, Tregs, neutrophils, and macrophages [63]. Chemokines are used by cancer cells to recruit immunosuppressive cells (e.g., TAMs), promote angiogenesis, and facilitate tumor growth, proliferation, and metastasis [64]. Elevated levels of chemokines, particularly C–C motif chemokine ligand 2 (CCL2), have been found in the TME. CCL2 exerts its activity through its receptor, C–C motif chemokine receptor 2 (CCR2), which is highly expressed by monocytes, DCs, and T cells [65]. In the TME, CCL2 activates Treg and inhibits CD8 + T effector cell activation [66]. CCL2 is often overexpressed by tumor cells, and CCR2/CCL2 overexpression has been associated with worse outcomes in multiple malignancies [65–67]. Blockade of this pathway may be used to enhance the effects of T effector cells and potentiate other forms of immunotherapy [66]. An area of concern is the unknown effects of its blockade in healthy tissues, given that the CCL2/CCR2 axis normally helps with infection control and facilitates wound healing [68].

PF-04136309, an oral inhibitor of CCR2, is being studied in a phase I clinical trial in combination with nab-paclitaxel and gemcitabine in patients with metastatic pancreatic adenocarcinoma (NCT02732938). Results of 21 patients revealed an ORR of 23.8% and a DCR of 38% with no CR, 5 confirmed PR, 1 unconfirmed PR, and 3 SD [69]. Response was indeterminate in 7 patients. Four patients had PD, 1 of which was an early death. DLTs included dysesthesia, hypokalemia, and hypoxia. There was a 24% incidence of pulmonary toxicities including three patients with grade 3 pneumonitis, one grade 4 hypoxia, and one grade 5 pneumonia. The authors concluded that the use of PF-04136309 was associated with worse pulmonary toxicities and no additional clinical benefit compared to gemcitabine and nab-paclitaxel alone [69]. The study was terminated early for administrative reasons and toxicity appears to be a concern based on preliminary data. Further exploration of this target in other cancers could be considered.

### LIF

Leukemia inhibitory factor (LIF) is a crucial peptide in embryogenesis. It promotes an immunosuppressive microenvironment that protects the embryo from the mother’s immune system, allowing its implantation and survival [70].

LIF also plays a role in cancer because it favors the immunosuppressive features of the TME by increasing CCL-2 and decreasing CXCL-9 release by TAMs. CXCL-9 is an important chemoattractant for cytotoxic CD8 + T cells [70]. LIF also enhances cancer cell proliferation, favors the development of a pro-invasive phenotype, and promotes chemotherapy and radiotherapy resistance [70]. Blocking this pathway could potentiate the effects of immunotherapy, chemotherapy, and radiotherapy. It is unclear whether the synergistic effects with existing therapy would come at the expense of increased immune toxicities. There is also concern that this therapy could affect pregnancy, particularly since low LIF levels have been associated with poor blastocyst implantation and infertility [71, 72].

MSC-1, a humanized IgG1 mAb targeting LIF, is being evaluated in a phase I clinical trial as monotherapy in patients with advanced, refractory solid malignancies (NCT03490669). Results available from 41 patients who received a median of 3 prior lines of therapy revealed a DCR of 22% with 9 patients achieving SD that lasted over 16 weeks [73]. In addition, tissue samples confirmed an increase in both M1:M2 ratio and cytotoxic CD8 + T cells. There were no DLTs. Although no grading is specified, the most common AEs included fatigue (20%) and gastrointestinal symptoms (20%). There was one patient with HNSCC who developed grade 2 osteonecrosis of the jaw; however, he had previously received radiation therapy to the area and had been exposed to denosumab [73]. Unfortunately, this trial was terminated early due to safety concerns. While the preliminary results were suggestive of clinical benefit, further research is needed to modify this agent to achieve better tolerability. If a safe and efficacious alternative is developed, perhaps this therapy could be combined with other agents in future trials.

### CD47/SIRPα

CD47 is a molecule expressed by nearly all normal tissue and serves as a marker of self-recognition. After binding the transmembrane protein ‘signal regulatory protein alpha’ (SIRPα) located on the surface of macrophages, CD47 prompts an anti-phagocytic signal [74, 75]. Under normal conditions, CD47 is under expressed in damaged cells to allow phagocytosis and tissue repair [75]. This molecule is often overexpressed in malignant cells, which blocks phagocytosis and favors metastatic dissemination. Overexpression of CD47 has been considered a poor prognostic factor in several malignancies including gastric, liver, lung, and BC [75–79].

Myeloid cells, including TAMs and DCs, are the most abundant type of cells in the TME. Inhibiting CD47 may boost macrophage phagocytosis against malignant cells [75, 80]. Additionally, the increase in antigen processing and presentation by DCs and TAMs indirectly leads to an enhanced tumor-specific cytotoxic T cell activity [81]. Anti-CD47 therapy may be safer than T cell-directed therapy because phagocytosis of cancer cells by macrophages would limit cancer cell content leakage [75]. The widespread expression of CD47 within normal tissues may limit its use. In particular, this therapy may be associated with red blood cell destruction and anemia [74, 82]. Lastly, higher or more frequent doses of therapy may be needed to achieve therapeutic blockade, an effect known as ‘antigen sink’ [74].

Hu5F9-G4 (5F9) is a humanized mAb that binds directly to CD47 and prevents its interaction with macrophages. One phase I clinical trial used this mAb as monotherapy in 43 patients with CRC, ovarian, adenoid cystic carcinoma, breast, pancreatic, and head and neck cancers (NCT02216409). The reported ORR was ~ 5% and DCR was 19% with 2 patients achieving PR (ovarian and fallopian tube cancers) and 6 SD (CRC) [83]. The median treatment duration was 18 weeks [83]. The most common AEs occurred with higher doses of therapy and included constitutional symptoms (50%), headache (34%), and hematological toxicities including anemia (39%) and lymphopenia (28%) [83]. This trial has been completed, and final publication is pending. Early results did demonstrate modest clinical benefit. Finals results will further evaluate the efficacy of this therapy. If the data are consistent with preliminary results and the toxicity is tolerable, then future research could evaluate the use of this therapy with other treatments in an attempt to increase response further.

Another phase I/II clinical trial evaluated Hu5F9-G4 combined with rituximab in relapsed and refractory non-Hodgkin's lymphoma (NHL) patients (NCT02953509). Data were available for 100 patients with a median age of 66 and a median of 3 prior lines of therapy [84]. Among the 75 evaluable patients, the CR rate (CRR) was 21% and ORR was 49% with 16 patients achieving CR and 21 achieving PR [85]. The median time to response was 1.8 months, and the median duration of response had not been achieved after 12 months of follow-up [84]. DLTs were reported in 4% of patients, but no specifics were provided. Grade 3 AEs consisted of anemia affecting 15% of patients. Most frequently reported AEs were limited to grade 1 and 2 and included infusion reactions (38%), gastrointestinal AEs (37%), headache (34%), and anemia (27%) [84]. This trial is active and recruiting. The available data suggest an impressive clinical response. These results support the use of this agent as an adjunct therapy. While this trial was limited to patients with NHL, future research can investigate whether this therapy is helpful in other malignancies.

ALX148 is a SIRPα fusion protein bound to an inactivated Fc domain that binds CD47 and results in blockade of both CD47-downstream signaling and its interaction with SIRPα on macrophages. A phase I clinical trial used this agent alone and in combination with pembrolizumab, trastuzumab, rituximab, ramucirumab, 5FU, paclitaxel, or cisplatin in patients with advanced solid malignancies or refractory NHL (NCT03013218). Preliminary results for 86 patients with HNSCC (*n* = 53) and gastric/gastroesophageal cancer (*n* = 33) were recently published [86]. Among patients with HNSCC, 52 received ALX148 with pembrolizumab and 1 patient received 5FU, a platinum, ALX148, and pembrolizumab. In this cohort, only 20 patients were evaluable for response. Ten were naïve to ICIs and 10 had received ICI therapy before. Among ICI-naïve patients, ORR was 40% (4/10), the median PFS was 4.6 months, and the median OS was not reached after 14 months of follow-up. Among the patients who were not ICI-naïve, the ORR was 0%, the median PFS was 2 months, and the median OS was 7.4 months [86]. Patients with gastric/gastroesophageal cancer received either ALX148 with trastuzumab (*n* = 30) or ALX148, trastuzumab, ramucirumab and paclitaxel (*n* = 3). Among patients who received ALX148 and trastuzumab alone that were response-evaluable (*n* = 20), ORR was 20%, median PFS was 2.2 months, and median OS was 8.1 months [86]. As a group, 82/86 patients experienced an AE; however, most (*n* = 57, 66.2%) were of low grade. The most common AEs included fatigue, elevated transaminases, cytopenias, and pruritus [86].

Another cohort sub-analysis from the same trial (NCT03013218) used ALX148 monotherapy in 25 patients with other solid malignancies [87]. DCR was 16% with 4 patients achieving SD, including 1 patient with NSCLC who had a 15% tumor reduction [87]. Twenty-two patients developed a toxicity. There were two DLTs consisting of neutropenia with infection and thrombocytopenia with a significant bleed. There was one grade 5 (fatal) toxicity that was under investigation. Four patients developed grade 3 and 4 toxicities including infection, pancreatitis, thrombocytopenia, and neutropenia. The other AEs were grade 1–2 [87]. This trial remains open and is actively recruiting. Final results will help assess clinical efficacy across a broad range of malignancies and provide comparison data. In addition, it will evaluate ALX148 as both adjunct and monotherapy. Information regarding the grade 5 toxicity will determine the future application of ALX148.

TTI-662 is another SIRPα fusion protein bound to an inactivated IgG4 Fc domain that targets CD47 and results in blockade of both CD47-downstream signaling and its interaction with SIRPα on macrophages. Unlike other anti-CD47 agents, TTI-662 does not bind to human erythrocytes and does not cause hemolysis [88]. A phase I clinical trial investigated this drug in patients with advanced relapsed or refractory lymphomas (NCT03530683). Results were recently published for use of this drug as monotherapy [88]. They included 19 patients (11 males, 8 females) with a median age of 62 years and a median of three previous lines of therapy. The authors reported 1 patient (diffuse large B cell lymphoma) who had received 5 prior lines of therapy and achieved a PR by week 8 and a CR by week 36 of treatment. There were no DLTs reported, and two patients developed grade 3–4 neutropenia. Grade 1 and 2 post-infusion thrombocytopenia was seen but was usually transient. No severe thrombocytopenia or anemia was reported [88]. This trial is actively enrolling. While preliminary clinical response is hard to assess given the limited data, the patient included had a remarkable response. If the final results demonstrate similar outcomes, this therapy may be a viable option for patients with refractory lymphoma. Further research could assess whether the addition of other therapies could augment TTI-662′s effect.

RRx-001 is a molecule that targets and downregulates both CD47 on cancer cells and SIRPα on macrophages [89]. A phase I clinical trial using this drug in combination with nivolumab in patients with advanced solid malignancies or lymphomas (NCT02518958) was completed. Results available for 12 patients at 12 weeks revealed an ORR of 25% and a DCR of 67% with 3 patients achieving PR, 5 SD, and 3 PD [90]. Although no DLTs were reported, one patient discontinued therapy due to pneumonitis and one voluntarily withdrew after a post-procedural infection. The most common AE related to RRx-001 was pain with the infusion (33%). Both pneumonitis (*n* = 1, 8.3%) and hypothyroidism (*n* = 1, 8.3%) were attributed to nivolumab [90]. This trial is closed, and final data analysis is pending publication. Early data suggests that this therapy is well tolerated and provided promising clinical response. The results will help assess the benefit of the addition of anti-CD47 and anti-SIRPα therapy to existing immunotherapy. If a benefit is seen, further research could evaluate the ideal ICI to use in combination with this therapy.

### CSF-1 (M-CSF)

As mentioned previously, TAMs are abundant in the TME. Under normal conditions, immature macrophages can differentiate into an active, pro-inflammatory, antitumor subtype (M1) or an immunosuppressive, pro-angiogenic, and pro-tumoral subtype (M2) [91]. In the TME, TAMs tend to express an M2 profile which favors tumor growth, angiogenesis, invasion, and early metastasis [92]. Increased number of TAMs within the TME correlates with poor prognosis [93].

TAM recruitment and differentiation into an M2 phenotype occur, in part, due to the interaction of colony-stimulating factor-1 (CSF-1 or macrophage-CSF [M-CSF]) with its receptor, colony-stimulating factor-1 receptor (CSF-1R or M-CSF-R) [94]. The latter is expressed by both TAMs and MDSCs. High levels of CSF-1R have also been associated with poor survival in several malignancies [95]. Blockade of the CSF-1 and CSF-1R interaction enhances the antitumor effects of immunotherapy and serves as an attractive therapeutic target [96]. It is unclear how big a role of the CSF-1/CSF-1R interaction plays in TAM activity. For this reason, it is also unclear what potential consequences this blockade will have or even how efficacious it will be [97]. It is also uncertain who will derive benefit from these therapies or what drug combination is most appropriate [95].

Lacnotuzumab (MCS110) is a recombinant mAb directed against CSF-1. It is being investigated in conjunction with spartalizumab in a phase I/II clinical trial in patients with advanced malignancies (NCT02807844). Preliminary results from 48 patients with melanoma, endometrial, pancreatic, and TNBC have been published [98]. The DCR was 27%, and 3 of the patients included had pancreatic cancer. One of them had a PR and 2 had durable SD lasting more than 300 days [98]. No additional details regarding response were provided. No DLTs were mentioned. There were some cases of grade ≥ 3 AEs including elevation of transaminases (12%) and hyponatremia (10%). Most AEs were grade 1 and 2 and included periorbital edema and elevated creatine kinase (CK) [98]. This trial was recently completed, and final results are pending publication. While clinical response was seen, further information is needed to assess efficacy and safety of this adjunct therapy. The trial evaluated ICI-naïve patients and those who had previously received this therapy. It will be interesting to evaluate the subset of melanoma patients who had been treated with ICIs. If response is favorable, perhaps lacnotuzumab can be used to augment response to ICIs.

LY3022855 is a human mAb against CSF-1R which is being studied in a phase I clinical trial as monotherapy in patients with metastatic BC and metastatic CRPC (NCT02265536). Results available for 34 patients (22 BC, 12 CRPC) were recently published [99]. In the BC group, there were no CR or PR but 1 had a noticeable reduction in a non-target neck mass. Only 7 of the CRPC patients were evaluable for response. ORR was 0% and DCR was 43% with 3 patients achieving SD lasting up to 4 months [99]. The severity of AEs was not available for review; however, some side effects included fatigue (38.2%), anorexia and nausea (26.5%, each), elevated lipase (23.5%), and elevated CK (20.6%) [99]. This trial was completed, and recently published data are consistent with the preliminary findings. Among the breast cancer population, the ORR was 0% and the DCR was 23% with no objective responses and 5/22 SD. Two of these responses lasted more than 9 months [100]. Future research can compare the response of anti-CSF-1R to anti-CSF-1 therapies. In addition, efforts could assess the efficacy of combination therapy with anti-CSF-1R, anti-CSF-1, and other immune therapies to improve clinical response.

SNDX-6352, another mAb targeting CSF-1R, is being evaluated in a phase I clinical trial as monotherapy and in combination with durvalumab in patients with refractory, advanced solid malignancies (NCT03238027). Results from 32 patients with a median age of 61 and a median of 5 lines of prior therapy demonstrated a DCR of 13%. Four patients achieved SD that lasted more than 4 months [101]. There were two patients who developed DLTs, one with grade 3 fatigue and one with grade 3 pneumonitis. Other grade 3 and 4 AEs were seen in 12 patients (38%) and included elevated CK (*n* = 5), elevated transaminases (*n* = 3), elevated amylase (*n* = 3), and elevated lipase (*n* = 2). Other non-severe AEs reported included periorbital edema (31%), nausea (13%), and anorexia (13%) [101]. This trial is active but not enrolling patients. Clinical response was modest; however, this was in the setting of heavily pre-treated disease. Finalized data will help determine safety of combination strategies and the benefit of monotherapy. Research utilizing alternative ICIs could be explored to determine the optimal combination therapy if safety profiles are satisfactory.

Emactuzumab (RG7155) is another humanized mAb that targets CSF-1R and is being studied as monotherapy or in combination with paclitaxel in a phase I clinical trial in patients with advanced or metastatic solid tumors (NCT01494688). Finalized results from 153 patients (99 treated with monotherapy, 54 with combination therapy) were published [102]. In the monotherapy cohort, ORR was 0% and DCR was 13% with 13 patients achieving SD. Based on the PET-CT results, 11 patients had a partial metabolic response and 40 had stable metabolic disease. Eighty-nine patients in the monotherapy group were unenrolled from the trial due to PD [102]. In the combination group, ORR was 7% with 4 patients (3 BC, 1 ovarian cancer) achieving PR, including 2 patients who had previously received a Taxane. DCR was 50% with 23 patients achieving SD. Based on the PET-CT results, there were 21 patients with partial metabolic response and 16 patients with stable metabolic disease. Forty-two patients in the combination group discontinued the trial due to PD [102]. Although no DLTs were reported in the monotherapy group, there were 2 DLTs in the combination cohort. One patient developed both grade 4 hypokalemia and grade 3 hemorrhagic enterocolitis, and one patient died (grade 5 AE) from a bowel perforation [102]. The authors concluded that emactuzumab did not result in clinically significant antitumor activity [102]. This trial has been completed. While the trial did not reveal any clinically significant benefit, perhaps combination with other agents may yield different results. Further research comparing the combination of anti-CSF-1R agents with alternative chemotherapy or immunotherapy may be beneficial.

Pexidartinib (PLX3397) is an oral inhibitor of the tyrosine kinase activity of CSF-1R that is being studied in combination with paclitaxel in a phase I clinical trial in patients with advanced solid malignancies (NCT01525602). The results of 54 patients were available for review [103]. Out of 38 patients evaluable, ORR was 16%, DCR was 50%, PD rate was 45% with 1 CR, 5 PR, 13 SD, and 17 PD. Two patients could not be assessed or lacked confirmatory scans. There were 2 DLTs: one grade 3 atrial fibrillation and one grade 3 hypophosphatemia. Grade 3 and 4 toxicities were seen in 38 patients (70%) and included cytopenias, elevated transaminases, and hypertension. Other AEs were grade 1 and 2 and included fatigue, anemia, and gastrointestinal toxicities [103]. This trial has been completed. Results are encouraging, and as outlined previously, assessment of optimal combination strategies are needed. In addition, it would be important to study differences among responders and non-responders to better select patients for this therapy. The oral administration of this drug is particularly attractive and convenient for patients.

Another phase I clinical trial evaluated the use of pexidartinib with durvalumab in patients with advanced or metastatic pancreatic adenocarcinoma or CRC (NCT02777710) [104]. Nineteen patients were included: 12 males and 7 females with a median age of 56 years. At 2 months, the ORR was 0% and the DCR was 21%. Four patients achieved SD, including 2 microsatellite unstable CRC patients whose response lasted more than 6 months [104]. There were 2 DLTs consisting of transaminase elevation, one which also included hyperbilirubinemia. Although no specific grading was provided, the most common AEs included rash, edema, and gastrointestinal toxicities. The most common grade 3 or 4 toxicities related to pexidartinib included cytopenias, elevated transaminases, and fatigue [104]. The trial has been completed, and final publication is pending. It is hard to assess the efficacy of this therapy given the limited results. In addition, 2 of the reported responses occurred in patients with microsatellite unstable disease, which is more likely to respond to PD-1/PD-L1 inhibitors like durvalumab [105]. Further analysis will help determine the added benefit of the pexidartinib therapy. Perhaps, future research could assess whether microsatellite unstable disease also serves as a marker of response to anti-CSF-1R therapy.

A phase I clinical trial evaluated pexidartinib monotherapy in Asian patients with symptomatic, advanced solid malignancies (NCT02734433). The results were available from 11 patients (6 males and 5 females) with a median age of 64 years. Among the 8 evaluable patients, the DCR was 67% with 1 PR and 4 SD [106]. The PR was ongoing at the time of cutoff at 7.6 months, and the mean duration of SD was 3.9 months. There were 3 patients with PD. Although all patients experienced an AE, 5 of them experienced at least one grade 3 or 4 AE. The most frequent grade 3 or 4 AEs reported were elevated transaminases and anemia [106]. This trial is ongoing but not actively enrolling. Clinical response was promising, and the therapy seemed to offer a reasonable side effect profile. Further research should evaluate the efficacy across a more diverse patient population as only Asian patients were included.

### IL-1 and IL-1R3 (IL-1RAP)

IL-1 was the first interleukin to be identified and is an important regulator of inflammation and innate and acquired immunity [107]. IL-1 has two basic isoforms, IL-1α and IL-1β. IL-1α is present in the cytoplasm of non-immune cell types and is released after cell death. IL-1β is released by DCs and macrophages in response to inflammation [108]. IL-1 (both α and β) exerts its function via the IL-1 receptor 1 (IL-1R1) expressed by T cells, B cells, NK cells, monocytes, macrophages, and DCs [108, 109]. IL-1R3 (also known as IL-1R accessory chain [IL-1RAP]) does not bind directly to IL-1, but it is recruited by the complex formed when IL-1 binds IL-1R1. It is essential for initiation of downstream signaling [109].

IL-1 overexpression in malignant cells contributes to chronic inflammation within the TME and T cell exhaustion [110, 111]. IL-1 promotes MDSC and TAM recruitment which further enhances immunosuppression, angiogenesis, and endothelial activation that favors tumor growth and metastasis [112, 113]. The IL-1 pathway has become an attractive therapeutic target. Blockade of this pathway can be achieved either by directly neutralizing IL-1 or by interfering with the IL-1R1 function (e.g., IL-1RAP inhibitor) [111]. An advantage of this pathway is that blockade can occur at multiple steps. Preliminary data suggest that this therapy is safe; however, data are unavailable regarding long-term toxicities. Given IL-1′s role in immune activation, the potential risk for infection is also of concern [111].

CAN04 is a first-in-class, fully humanized, mAb that targets IL-1RAP and blocks IL-1 α and β signaling. A phase I clinical trial using CAN04 as monotherapy in patients with advanced or metastatic NSCLC, CRC, BC, or pancreatic adenocarcinoma is being conducted (NCT03267316). Results from 22 patients (14 males and 8 females) with a median age of 62 years and a median of 3 prior lines of therapy were available [114]. Among the 20 patients evaluated, the DCR was 45% with 9 SD including 2 (1 NSCLC and 1 pancreatic adenocarcinoma) whose response lasted more than 4 months. No DLTs or grade 4–5 AEs were reported. There were three grade 3 AEs including one infusion reaction, one leukopenia, and one hypokalemia. The most common AEs were grade 1–2 and included infusion-related reactions (41%), fever (27%), chills (23%), and nausea (23%) [114]. This trial is open and recruiting, and additional research will help confirm clinical efficacy and tolerability. In the future, efforts can evaluate combination therapy to further improve response. In addition, the application of this therapy could be explored in hematologic malignancy.

Canakinumab (ACZ885), a recombinant human IgG mAb, binds and blocks IL-1β and is being investigated as monotherapy and in combination with pembrolizumab in a phase II clinical trial in patients with early-stage NSCLC (NCT03968419). No results are available yet. The trial is open for enrollment.

### IL-8

IL-8, also known as CXCL8, is an inflammatory chemokine that mediates its effects via IL-8R-A and B (also known as CXCR1 and 2) [115]. Under normal conditions, IL-8 is produced by monocytes, endothelial cells, and epithelial cells in response to infection or tissue injury. It plays a role in neutrophil recruitment and promotion of angiogenesis to facilitate healing [116].

The IL-8/IL-8R axis is overexpressed in solid and hematologic malignancies (e.g., breast, ovarian, lung, Hodgkin’s lymphoma). It promotes angiogenesis and facilitates oncogenic signaling, invasion, and resistance [116]. IL-8 also induces an immunosuppressive TME by recruiting MDSCs [116]. Elevated levels of IL-8 correlate with worse outcomes and ICI resistance [117]. Inhibition of IL-8 and its receptors are a promising target in immunotherapy. One potential limitation of this therapy is the effect it may have on angiogenesis and immune response, particularly in response to tissue injury and infection [115].

BMS-986253, a fully human anti-IL-8 mAb, is being studied as monotherapy in a phase I clinical trial in patients with metastatic or unresectable solid tumors (NCT02536469). Preliminary results available for 15 patients demonstrated an ORR of 0% and a DCR of 73% with 11 cases of SD and 4 PD [118]. PFS was 73% at 24 weeks. Although no serious or life-threatening AEs were reported, 33% (*n* = 5) developed mild constitutional symptoms, hypersomnia, or mild hypophosphatemia [118]. The trial is completed, and finalized results are consistent with the preliminary data. The final PFS was 53.3% at 5.5 months, and no grade ≥ 3 AEs were reported [119]. Further research is needed to assess its use in practice. Perhaps combination therapy will provide improved clinical response.

Another phase Ib/II clinical trial is evaluating BMS-986253 in combination with nivolumab in patients with advanced solid malignancies (NCT03400332). No preliminary results are available. The study is active but not enrolling. The results will help to determine whether the addition of existing ICIs improves clinical response to BMS-986253.

### Semaphorins/SEMA4D

Semaphorins are a family of transmembrane proteins that assist with axonal repair after neuronal injury, cytoskeletal changes, and migration of endothelial and immune cells [120]. They also play a role in modulating immunity and angiogenesis in the TME as well as favoring cancer cell survival and metastasis [121]. Among this family, SEMA3A, SEMA3B, and SEMA4D have all been implicated in the recruitment of TAMs to the TME, and they promote an immunosuppressive microenvironment [121].

SEMA4D (CD100) binds 3 types of receptors, Plexin-B1 (PLXNB1), Plexin-B2 (PLXNB2), and CD72, which are all expressed by APCs (B cells, monocytes, DCs), endothelial cells, and tumor cells [122]. Upon binding to its receptor, SEMA4D promotes activation and migration of endothelial cells and tumor cells. It blocks immune infiltration of active T cells and favors a shift toward Tregs. It also increases the levels of monocyte chemoattractant protein 1 (MCP-1), the most important chemokine for macrophage recruitment and differentiation into TAM/M2 phenotype in the TME [122, 123]. The use of anti-SEMA4D agents has shown to revert these effects and leads to an increased number of active immune cells within the TME [122]. Anti-SEMAD4D agents have been used in conjunction with ICIs to improve response in those who failed prior ICI therapy. Although the safety profile has been acceptable, there is a risk for “on-target, off-tumor” effects and immunological defects given its widespread expression in normal tissue [122]. Lastly, given the role of semaphorins in the nervous system, there is a theoretical risk of neurotoxicity, particularly in developing or injured neuronal tissue.

Pepinemab (VX15/2503), a humanized IgG4 monoclonal antibody against SEMA4D, is being studied in a phase I/II clinical trial in combination with avelumab in patients with advanced-stage NSCLC (NCT03268057). Preliminary results from 62 patients (only 50 were evaluable for response) were recently published [124]. Among 21 evaluable patients who were immunotherapy naïve, the ORR was 24% and the DCR was 81% with 5 patients achieving PR and 12 SD. There were 3 of these patients whose benefit extended beyond 1 year. A subgroup analysis revealed that patients who had a negative or low PD-L1 expression had an ORR that was twofold–2.5-fold greater with combination therapy than with single ICI agents. In fact, 97% of patients with PR or SD had tumors with negative or low PD-L1 expression [124]. Among 29 evaluable patients of the ICIs-refractory group, the ORR was 7% and the DCR was 59% with 2 patients achieving PR and 15 SD. There were 7 patients in whom the clinical benefit extended beyond 23 weeks. Although no specific details about AEs were available, no AEs led to drug discontinuation or death [124]. The trial has been completed, but the final results are pending publication. Overall clinical response to therapy was promising, particularly in tumors with negative or low PD-L1 expression and those treated with combination therapy. Perhaps additional research will support the use of anti-SEMA4D agents as adjunct therapy in patients with low PD-L1 expression with NSCLC and other malignancies.

### Ang-2

Angiopoietins 1 (Ang-1) and 2 (Ang-2) are growth factors that help maintain vascular integrity and play a role in vascular homeostasis and growth [125]. Although both molecules act on the same receptor, Tie2, they have opposite effects. Ang-1 promotes vessel stability, but Ang-2 disrupts the vascular integrity while promoting sprouting and endothelial cell apoptosis [126]. Ang-2 also plays a role in inflammation by facilitating myeloid cell adhesion and trafficking, increasing capillary leakage, and inducing monocyte polarization into M2/TAMs phenotype that releases anti-inflammatory cytokines and recruits Tregs [126, 127]. Ang-2 is overexpressed in the TME and tumor vasculature. High levels of Ang-2 correlate with worse outcomes in many malignancies [128]. Blockade of this pathway could simultaneously affect two pro-tumorigenic pathways: angiogenesis and inflammation [126]. Additionally, increased Ang-2 levels are seen in patients with ICI-resistant disease. Therapy targeting this pathway could also help patients with ICI-resistant cancers [128]. It remains unclear whether the use of anti-Ang-2 molecules will impact non-tumor tissues. If it does, it is also unclear what angiogenic and immunologic implications would arise from this therapy.

Trebananib, an anti-Ang-1/Ang-2 neutralizing antibody that interferes with Tie-2 activation, is being studied in combination with pembrolizumab in a phase I clinical trial in patients with advanced solid tumors (NCT03239145). Results from 18 heavily pretreated patients with microsatellite stable (MSS) CRC and a median age of 51 years were recently published [129]. In the 15 evaluable patients, DCR was 33% and ORR 7% with 1 patient achieving PR and 4 SD. Median time to progression was 2.6 months, and OS was 11.4 months. There were no DLTs, and no grade 3 or 4 AEs attributed to trebananib. Other reported AEs included abdominal distention, diarrhea, edema, and proteinuria, each occurring in 40% of patients [129]. The trial remains open and is currently recruiting patients. While results are only available for MSS CRC patients, the results are suggestive of some benefit to combination therapy with pembrolizumab and trebananib. A previous clinical trial reported an ORR of 0% and a PFS of 11% among MSS CRC patients receiving pembrolizumab monotherapy [130]. Additional studies will be needed to confirm these findings and determine the benefit of this combination.

### CLEVER-1

Common lymphatic endothelial and vascular endothelial receptor-1 (CLEVER-1) is a scavenger receptor that is found on endothelial cells and tissue-M2 phenotype macrophages [131]. This molecule mediates cellular adhesion and cell trafficking, and it has also been linked with immune modulation mediated by M2 macrophages [132]. CLEVER-1 is expressed by TAMs. Elevated CLEVER-1 levels have been associated with poor prognosis in certain malignancies [132]. Blockade of the pathway results in a reduced number of CLEVER-1-expressing TAMs. It also induces an M1 macrophage phenotype in the TME and reactivates and recruits CD8 + T cells [132]. Anti-CLEVER-1 agents could be effective when combined with other ICIs, particularly in aggressive, non-responsive tumors and even in the so-called cold tumors [132]. The ideal sequence of therapy is uncertain. It is unclear whether this therapy should be used before or after immunotherapy in an attempt to turn “cold tumors” into “hot tumors.” Additionally, the ideal combination of therapies and long-term toxicities, particularly given its role in endothelial cell function, are unknown.

FP-1305, a humanized IgG4 mAb targeting CLEVER-1, is being studied as monotherapy in a phase I/II clinical trial in patients with advanced and refractory solid malignancies (NCT03733990). Preliminary results are available for 30 patients (22 females and 8 males) with a median age of 65 years [133]. ORR was 3% and DCR was 27% with 2 patients achieving PR (one was a heavily treated CRC), 6 SD, and 22 PD. At the time of cutoff, only 1 patient remained in the study, and the other 29 patients had been unenrolled due to PD or provider’s choice [133]. Patients’ serum Treg levels decreased, and CD8 + T cells and NK cells were increased compared to baseline. There were no DLTs. Although 67% of patients experienced an AE, there was only one grade 3–4 AE (infected seroma). One individual developed serious AEs including pneumonitis (grade 1), dermatitis, myositis, and thyroiditis (each grade 2). The most common grade 1 and 2 AEs included fatigue, fever, elevated transaminases, and gastrointestinal toxicities [133]. This trial is active and recruiting. The preliminary data suggests a potential change in the TME from a “cold” to “hot” TME using this therapy. If this finding is confirmed, perhaps this will allow for a better response to other immune therapies. As mentioned above, the sequence of therapy or need for upfront combination treatment could also be explored.

### Axl

Axl (also known as UFO, ARK, Tyro7, or JTK11) is a tyrosine kinase receptor member of the TAM (Tyro3/Axl/Mer3) receptor family. It is generally expressed by platelets, endothelial, cardiac, hepatic, nervous, and immune cells such as monocytes, macrophages, NKs, and DCs [134]. After binding to its ligand, Gas6, Axl promotes cell survival, activates phagocytosis, and induces an immunosuppressive phenotype in DCs, macrophages, and NK cells [134, 135]. In addition to its direct immunosuppressive effects, Axl decreases antigen presentation and increases immunosuppressive cytokines, indirectly interfering with T cell activation [136].

Axl also plays a role in the development of cancer. It favors cancer cell proliferation and promotes resistance to chemotherapy, targeted therapy, radiation therapy, and immunotherapy. Axl enables malignant cell migration and invasion. This is made possible by Axl’s ability to regulate vessel growth and induce an epithelial-to-mesenchymal transition (EMT) which favors the development of the malignant cell invasive phenotype [135]. Axl is upregulated in hematologic and solid malignancies and is associated with poor prognosis [134].

Axl may have therapeutic benefit, and agents targeting the receptor or its tyrosine kinase activity are being developed [135]. Blockade of Axl may result in “on-target, off-tumor” toxicities given its widespread expression. It is uncertain whether these agents are potent enough to be used as monotherapy. If they must be combined, it is unclear which agents to use [135].

Enapotamab vedotin (EnaV) is an ADC formed by an IgG1 mAb against Axl and a microtubule inhibitor. It is being evaluated as monotherapy in a phase I, first-in-human clinical trial, in patients with relapsed, refractory solid malignancies (NCT02988817). Preliminary results from 47 patients, predominately female (87%) with a majority age less than 65 were published [137]. The reported ORR was 6%. The DCR was 55% with 3 patients achieving PR and 26 SD. There were 6 DLTs including constipation, vomiting, GGT elevation, febrile neutropenia, and diarrhea. Forty-six patients experienced an AE. Thirty-one cases were grade 3 and 4 AEs including fatigue, diarrhea, and vomiting. The most common grade 1 and 2 AEs included fatigue, nausea, constipation, and poor appetite [137]. This trial remains open for enrollment. Initial clinical response is encouraging; however, toxicity is a concern. Further research could expand the use of this therapy to include hematologic malignancy.

### Phosphatidylserine

Phosphatidylserine (PS) is a phospholipid located in the inner layer of the plasma membrane of eukaryotic cells. Once the cell dies, the PS is moved to the outer layer of the membrane and is exposed [138].

PS receptors (PSR) are a family of receptors expressed by endothelial cells, MDSCs, macrophages, DCs, B, T, and NK cells. PSRs can directly or indirectly (with the help of bridging proteins) bind PS [138]. Examples of PSR include the TIM receptors (directly bind PS) and TAM receptors (indirectly bind PS) [138]. The PS/PSR interaction triggers efferocytosis and activates inhibitory pathways that prevent the development of inflammation in response to apoptosis [138].

PS is overexpressed by tumor and endothelial cells by the tumor vasculature and TME. Levels are further increased with chemotherapy and radiation exposure that result in cancer cell death and PS release [139]. Targeting this pathway, either by blocking PS directly or the PSRs (e.g., TIM or TAM receptors as discussed above), can enhance immune response against the tumor and potentiate the effects of chemotherapy and radiotherapy [138]. Use of this therapy could be limited given the widespread PSR expression and potential effects of its blockade in healthy tissue.

Bavituximab, an IgG3 mAb against PS, is being investigated in combination with sorafenib in a phase II clinical trial in patients with advanced, unresectable hepatocellular carcinoma (HCC) (NCT01264705). Results from 38 patients with a median age of 61 were available for review [140]. The ORR was 5%. The DCR was 58% with 2 patients achieving PR and 20 SD. The median time to progression was 6.7 months, and the median OS was 6.1 months. Although this was better than historical controls, the primary endpoint of median time to progression of 8.2 months was not met, and thus, results were deemed inconclusive [140]. There were no DLTs, or grade 4–5 AEs reported. The reported incidence of AEs was 63%, all were grades 1–3 and included diarrhea (32%), fatigue (26%), and anorexia (24%) [140]. The trial was completed, and final results are pending publication. Unfortunately, the preliminary results did not meet their primary endpoint. The study was limited to HCC, and it is unclear how other malignancies might respond to this therapy.

## Conclusion

Immunotherapy revolutionized oncology and improved outcomes and survival for many cancer patients. ICIs targeting CTLA-4 and PD-1/PD-L1 have become a cornerstone in the management of malignancy. Unfortunately, response to ICIs remains low. In an attempt to improve response to immunotherapy, additional agents and inhibitory pathways are being explored, but their development remains a challenge. Often, these novel therapies are not potent enough to be used alone but can potentiate the effects of existing therapy. This synergism may result in an increased incidence and severity of immune-related AEs. New toxicities including ‘on-target off-tumor’ effects have been described, and the effects of these therapies on healthy tissue remains a concern. Future research is needed to identify biomarkers that could help select patients who may benefit the most while also avoiding significant toxicities. Many of these therapies lack activity in ‘cold’ TMEs. Strategies to promote a switch to ‘hot’ TMEs may enhance the efficacy and expand the application of these therapies. Despite these challenges, immune checkpoint inhibitors remain a vital and promising tool in the fight against cancer.

## Data Availability

All data generated or analyzed during this study are included in this published article.

## Abbreviations

AE: Adverse event
Ang: Angiopoietin
APCs: Antigen-presenting cells
ADC: Antibody-drug conjugate
ATP: Adenosine triphosphate
BC: Breast cancer
CCL-2: C–C motif chemokine ligand 2
CCR: C–C motif chemokine receptor
CEACAM: Carcinoembryonic antigen-related cell adhesion molecule
CK: Creatine kinase
CLEVER-1: Common lymphatic endothelial and vascular endothelial receptor-1
CR: Complete response
CRC: Colorectal cancer
CRPC: Castrate-resistant prostate cancer
CRR: Complete response rate
CRS: Cytokine release syndrome
CSF-1: Colony-stimulating factor-1
CTLA-4: Cytotoxic T lymphocyte-associated molecule-4
DCs: Dendritic cells
DCR: Disease control rate
DLBCL: Diffuse large B cell lymphoma
DLTs: Dose-limiting toxicities
EMT: Epithelial-to-mesenchymal transition
GGT: Gamma-glutamyl transferase
HCC: Hepatocellular carcinoma
HNSCC: Head and neck squamous cell carcinoma
ICI: Immune checkpoint inhibitor
IL-1RAP: IL-1 receptor accessory chain
irAE: Immune-related adverse event
LAG3: Lymphocyte activation gene-3
LIF: Leukemia inhibitory factor
M-CSF: Macrophage-CSF
mAb: Monoclonal antibody
MDSCs: Myeloid-derived suppressor cells
MHC: Major histocompatibility complex
mRNA: Messenger RNA
MSS: Microsatellite stable
NHL: Non-Hodgkin's lymphoma
NKs: Natural killers
NKG2A: Natural killer group protein 2A
NSCLC: Non-small cell lung cancer
ORR: Objective response rate
OS: Overall survival
PD: Progressive disease
PD-1: Programmed cell death receptor-1
PD-L1: Programmed cell death receptor-1 ligand
PFS: Progression-free survival
PI3K: Phosphoinositide 3-kinase
PR: Partial response
PS: Phosphatidylserine
PVRIG: Poliovirus receptor-related immunoglobulin domain-containing
PVRL2: Poliovirus receptor ligand
RCC: Renal cell carcinoma
RFS: Recurrence-free survival
SCLC: Small cell lung cancer
SD: Stable disease
SIRP: Signal regulatory protein
SMAC: Second mitochondrial activator of caspase
STING: Stimulator of interferon genes
SYK: Spleen tyrosine kinase
TAMs: Tumor-associated macrophages
TILs: Tumor-infiltrating lymphocytes
TIM: T cell immunoglobulin
TLR: Toll-like receptor
TME: Tumor microenvironment
TNBC: Triple-negative breast cancer
TNF: Tumor necrosis factor
Tregs: Regulatory T cells
